# Clerodane Furanoditerpenoids from *Tinospora bakis* (A.Rich.) Miers (Menispermaceae)

**DOI:** 10.3390/molecules29010154

**Published:** 2023-12-26

**Authors:** Ahmed Saeed Kabbashi, Maazah Abdul Sattar, Muhammad Aamer, Nimra Naz Siddiqui, Muhammad Kamran, Aneela Fayaz, Humera Jahan, Farooq-Ahmad Khan, Yan Wang

**Affiliations:** 1 H. E. J. Research Institute of Chemistry, International Center for Chemical and Biological Sciences, University of Karachi, Karachi 75270, Pakistan; ahmedsak88@gmail.com (A.S.K.); maazahmughal@gmail.com (M.A.S.); m.aamer00786@gmail.com (M.A.); mkchemist785@gmail.com (M.K.); aneelafayaz1993@gmail.com (A.F.); 2 Department of Microbiology, Faculty of Pure and Applied Sciences, International University of Africa, Khartoum 12223, Sudan; 3 Dr. Panjwani Center for Molecular Medicine and Drug Research, International Center for Chemical and Biological Sciences, University of Karachi, Karachi 75270, Pakistan; nazsiddiqui92@gmail.com (N.N.S.); jahan_pcmd@yahoo.com (H.J.); 4 Third World Center (TWC) for Chemical Sciences, International Center for Chemical & Biological Sciences, University of Karachi, Karachi 75270, Pakistan

**Keywords:** diabetes, furanoditerpenoids, tinobakiside, tinobakisin, *Tinospora bakis*

## Abstract

*Tinospora bakis* (A.Rich.) Miers (Menispermaceae) has traditionally been used to alleviate headaches, rheumatism, mycetoma, and diabetes, among others. Despite its extensive use, the active components of the plant have never been investigated. In this work, a series of furanoditerpenoids (**1**–**18**) and five compounds from other classes (**19**–**23**) were isolated from *T. bakis*. Notably, two new compounds were discovered and named: tinobakisin (**1**) and tinobakiside (**10**). Their molecular structures were elucidated with NMR, MS, UV, IR, and ECD spectra. Additionally, known compounds (**2**–**9** and **11**–**23**) were corroboratively identified through spectral comparisons with previously reported data, while highlighting and addressing some inaccuracies in the prior literature. Remarkably, compounds **6**, **7**, **13**, and **17** exhibited a superior anti-glycation effect, outperforming established agents like rutin and quercetin in a lab model of protein glycation with glucose. The overall findings suggest that furanoditerpenoids play a crucial role in the antidiabetic properties of *T. bakis.* This research marks the first comprehensive phytochemical investigation of *T. bakis*, opening the door for further investigation into furanoditerpenoids and their biological mechanisms.

## 1. Introduction

Diabetes has been a severe health and economic burden globally, with increasing prevalence year by year. It is worth mentioning that around 422 million adults were living with diabetes in 2014 globally, in comparison to 108 million in 1980, and the prevalence is expected to approach 629 million worldwide in 2045 [1,2]. Therefore, treating diabetes and its associated comorbidities has been a hot field, attracting the attention and effort of scientists [3]. Natural products have been indispensable sources for new drug discovery. Among the approved drugs from 1981 to 2014, natural products or their derivative accounted for 26% [4]. Folk herbal medicines have been used worldwide for controlling blood glucose levels in patients with diabetes since ancient times [5]. Many investigations have been conducted to explore the active components for advanced antidiabetic drug discovery [6].

The *Tinospora* genus, belonging to the family Menispermaceae, has 16 accepted species (World Flora Online). The species are widely distributed throughout the tropical and subtropical parts of Asia, Africa, and Australia. Several species, such as *T. capillipes*, *T. cordifolia*, *T. sagittata*, *T. sinensis*, etc., have been well-studied due to their medicinal importance. Until now, more than 200 secondary metabolites have been isolated from these species, including diterpenoids, triterpenoids, sesquiterpenoids, alkaloids, steroids, flavonoids, lignans, etc. [7,8,9], among which more than 100 compounds belong to clerodane furanoditerpenoids [10,11,12,13,14,15,16].

*Tinospora bakis* (A.Rich.) Miers is a deciduous climber, wildly growing in Africa. Its whole plant, root, or leaves have been used to treat headaches, rheumatism, mycetoma, diabetes, etc., in Africa [8,17,18]. Despite its therapeutic importance, the phytochemicals of *T. bakis* have never been investigated, except for the main component, columbin [17]. Although the extracts from the roots or whole plant of *T. bakis* have been studied through in vivo pharmacological approaches, demonstrating antipyretic, antidiabetic, and immunomodulatory effects, most of the active components in *T. bakis* remain unknown [18,19,20].

**Figure 1:**
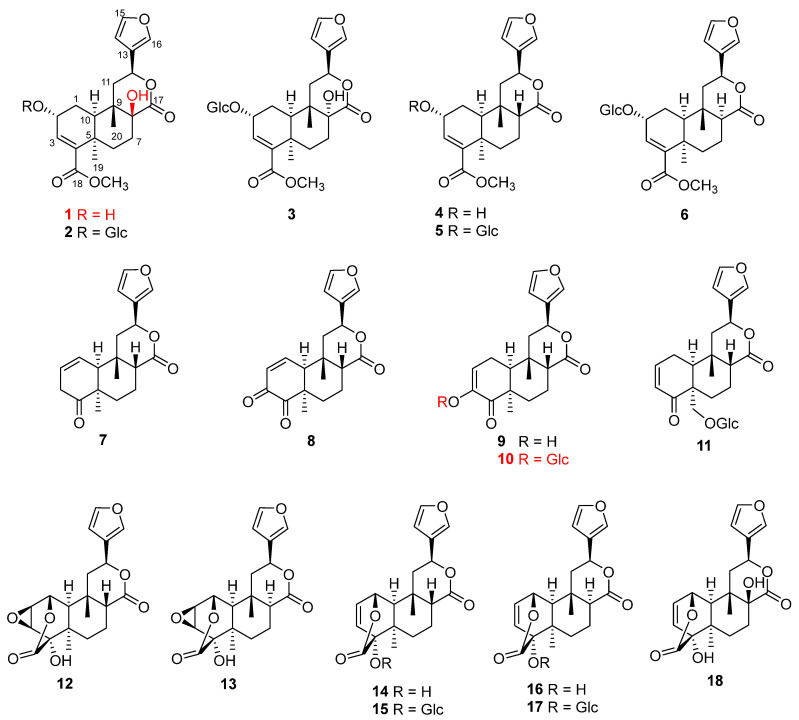
Structures of compounds **1**–**18**.

In this work, a systematic phytochemical investigation was conducted on *T. bakis* for the first time. A total of 23 compounds were isolated from the EtOAc fraction of *T. bakis.* The structures of all compounds were elucidated based on multiple pieces of spectroscopic evidence, such as NMR, MS, UV, IR, and ECD. Among them, 18 compounds (**1**–**18**) were furanoditerpenoids, as shown in Figure 1. Moreover, an in vitro anti-glycation essay on the obtained components was performed, resulting in several potential antidiabetic leads, which shed light on the molecular basis of the antidiabetic property of *T. bakis*. Further investigation of the in vivo evidence of the active components and related mechanisms might be required [21,22,23].

## 2. Results and Discussion

**Figure 2:**
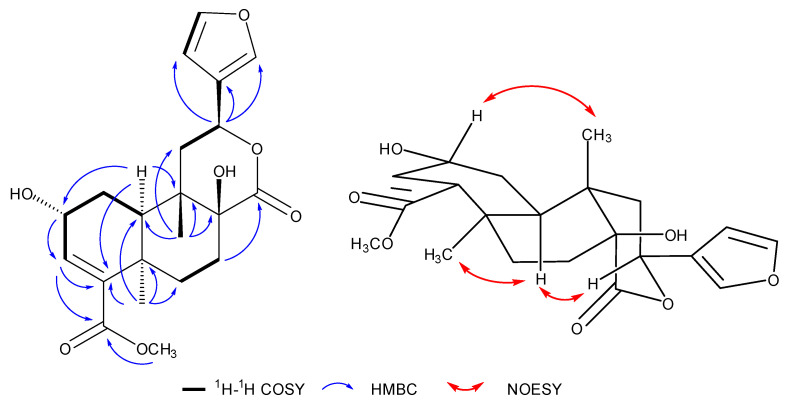
Key ^1^H–^1^H COSY, HMBC, and NOESY correlations of compound **1**.

**Figure 3:**
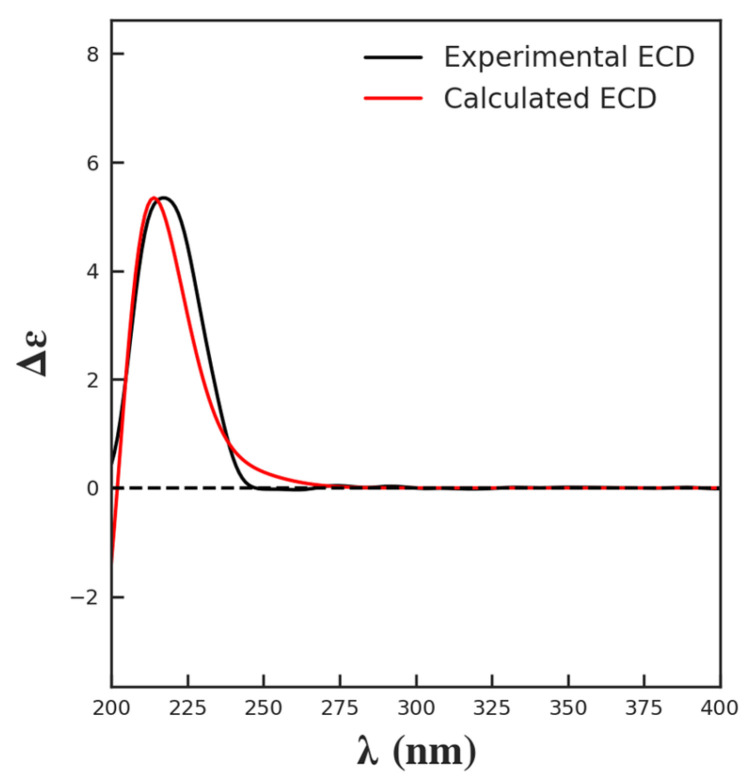
Experimental and calculated ECD of compound **1**.

Compound **1** was obtained as white needle-like crystals. The molecular formula was deduced as C_21_H_26_O_7_ according to the molecular ion peak [M]^+^ at *m*/*z* 390.1687 (calcd. for 390.1679) in HR-EI-MS, with nine unsaturation degrees. The ^1^H-NMR data (Table 1) displayed the characteristic signals for a furan ring at *δ*_H_ 7.54 (br s), 7.39 (t, *J* = 1.2 Hz), and 6.73 (d, *J* = 1.2 Hz). An olefinic proton at *δ*_H_ 6.57, two oxymethine signals at *δ*_H_ 5.15 (dd, *J* = 11.2, 4.8 Hz) and 4.46 (m), two methyls at *δ*_H_ 1.44 (s) and 1.05 (m), and a methoxy at *δ*_H_ 3.73 were also observed. The ^13^C-NMR (Table 1) and DEPT spectra revealed the existence of 21 carbons, including two carbonyl signals at *δ*_C_ 178.3 and 169.5; 4 carbons for the furan group at *δ*_C_ 144.1, 142.0, 127.6, and 110.9; 2 olefinic carbons at *δ*_C_ 141.1 and 140.4; 3 oxygen-substituted alkyl carbons at *δ*_C_ 90.4, 74.3, and 65.1; two methyls at *δ*_C_ 30.6 and 22.2; and a methoxy at *δ*_C_ 52.1, along with another 2 quaternary carbons, one methine, and four methylenes in the upfield. All the data agreed with the aglycon of tinospinosides B (**2**) [11]. The structure was further confirmed by correlations in the ^1^H–^1^H COSY, HSQC, and HMBC spectra, which are assigned in Figure 2. Correlations of H-2 (*δ*_H_ 4.46)/CH_3_-20 (*δ*_H_ 1.05), H-10 (*δ*_H_ 1.92)/CH_3_-19 (*δ*_H_ 1.44), and H-10 (*δ*_H_ 1.92)/H-12 (*δ*_H_ 5.15) in the NOESY spectrum indicated the configurations of chiral carbons. Since enantiomers of clerodane diterpenoids commonly exist naturally [24], the relative configuration of **1** was also further proved by curve fitting the experimental and calculated ECD. As shown in Figure 3, the calculated ECD of the proposed structure showed an excellent agreement with the experimental ECD of **1**. Therefore, the structure of compound **1** was confirmed and named tinobakisin.

Compounds **2** and **3** were identified as tinospinoside B and tinospinoside C, respectively [11]. Compound **4** was found identical to the aglycon of tinospinoside A (**5**), obtained after hydrolysis. Therefore, it is isolated as a natural compound for the first time herein [11]. Compound **6** was determined as the 8-epimer of tinospinosides A, tinophylloloside [25]. Compounds **7**–**9** were identified as tinocallone A [26], fibaruretin H [27], and sagitone [14], respectively.

**Figure 4:**
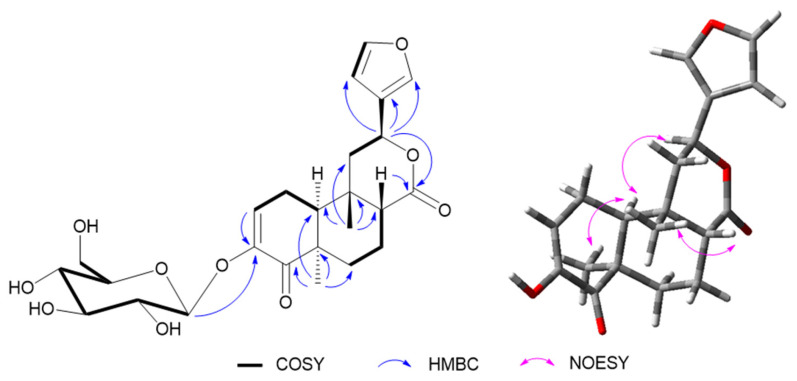
Key ^1^H–^1^H COSY, HMBC, and NOESY correlations of compound **10**.

**Figure 5:**
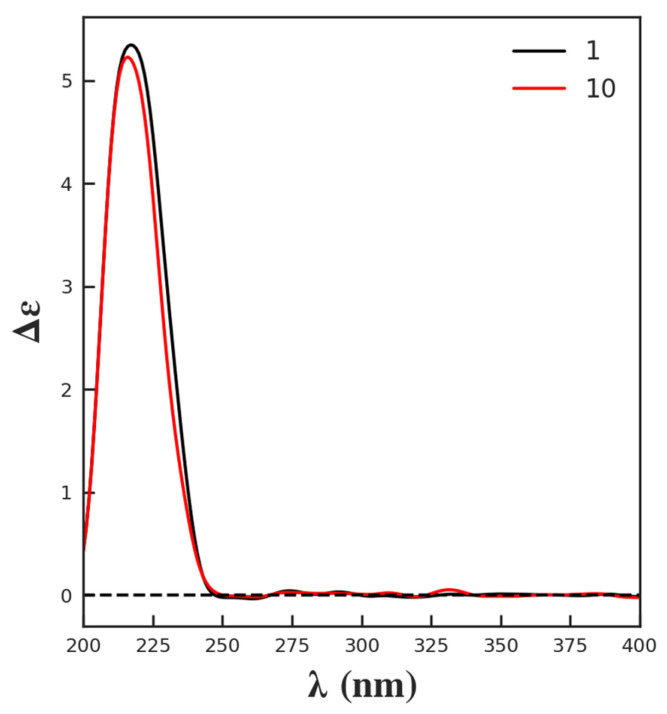
Experimental ECD of compounds **1** and **10**.

Compound **10** was isolated as an amorphous, colorless semisolid. The molecular formula was deduced as C_25_H_32_O_6_, through the molecular ion peak [M + H]^+^ at *m*/*z* 493.2061 (calcd. for 493.2074, C_25_H_33_O_6_) in positive HR-FAB-MS, suggesting 10 degrees of unsaturation. The ^1^H-NMR data (Table 1) displayed the characteristic signals for a furan moiety at *δ*_H_ 7.59 (br s), 7.49 (t, *J* = 2.0 Hz), and 6.54 (d, *J* = 2.0 Hz); an olefinic proton at *δ*_H_ 6.30 (dd, *J* = 6.5, 2.5 Hz); an oxymethine signal at *δ*_H_ 5.57 (dd, *J* = 12.5, 4.0 Hz); and two methyls at *δ*_H_ 1.28 (s) and 0.97 (s). ^13^C-NMR (Table 1) revealed the presence of two carbonyl carbons at *δ*_C_ 200.5 and 174.8; aromatic carbons at *δ*_C_ 149.2, 145.0, 141.4, 126.8, 124.3, and 109.6; and two angular methyls at *δ*_C_ 29.0 and 26.6. All these data of **1** were similar to those of sagitone, a novel 18-norclerodane furanoditerpenoid isolated from the roots of *Tinospora sagittata* var. *yunnanensis* [14]. However, additional signals belonging to a glucopyranoside at *δ*_C_ 102.3, 74.6, 78.3, 71.3, 77.6, and 62.5, corresponding to *δ*_H_ 4.62, 3.84, 3.65, 3.39, 3.38, 3.34, and 3.32 in the HSQC spectrum, were also observed [28]. After acid hydrolysis, the sugar moiety was determined as D-glucose, based on the optical rotation detected by an optical detector through HPLC. Also, the large coupling constant exhibited by the anomeric proton at *δ*_H_ 4.62 (1H, d, *J* = 7.0 Hz) suggested the relative configuration as β-oriented (Figure 4). In addition, the experimental ECD of **10** was close to **1** (Figure 5), indicating a similar relative configuration. Thus, the structure of **10** was established and named tinobakiside.

Compound **11** was elucidated as palmatoside G [29]. Compounds **12** and **13** were determined to be jateorin and isojateorin, respectively, based on the NMR data [30,31]. The X-ray structure of jateorin was reported in 1986 as a major component of *Tinospora cordifolia* [32]. Tinosporide was previously wrongly identified from *Tinospora cordifolia* and then later confirmed by X-ray to be identical to jateorin [33]. Chasmanthin and palmarin from *Jateorrhiza palmate*, *Jateorhiza palmate*, and *Fibraurea chloroleuca* were reported as the 12-epimer of jateorin and isojateorin, respectively [30,31,34], and also found in *Tinospora cordifolia* [35], which proved the common existence of these compounds in the Menispermaceae family. The structure of **14** was initially deduced, as reported by Hanuman et al. [36], because the NMR values were identical. However, after careful elucidation by 2D NMR, it was determined as the columbin, which was also isolated from several other species of the *Tinospora* genus [14,16,37,38] and found to be the major component of *T. bakis* [17]. This literature might wrongly determine the structure [36] in which 2D NMR was not performed. In addition, this is the only report of this structure. Compound **14** had β-H at C-8. Compound **15** had α-H (*δ* 2.98) at C-8 as its isomer [30]. Compounds **16** and **17,** palmatoside C and D, the glucosides of **14** and **15**, were isolated together from *Jateorhiza palmata* Miers in 1987 [29]. Later, palmatoside C was also found in the *Tinospora* genus [14,37]. Its configuration can be confirmed by the correlations with CH_3_-20 (δ 1.26) and H-10 (*δ* 1.84) in the NOESY spectrum. Compound **18** was elucidated as 8-hydroxycolumbin [39]. 

Five compounds from other classes were elucidated, based on their NMR and MS data, as 4-[formyl-5-(hydroxymethyl)-1*H*-pyrrol-1-yl] butanoic acid (**19**) [40], quercetin (**20**) [41], β-sitosterol (**21**) [42], β-sitosterol β-D-glucoside (**22**) [43], and oleic acid (**23**) [44].

The anti-glycation activities of all clerodane furanoditerpenoids **1**–**18** were evaluated by the in vitro BSA (bovine serum albumin)–glucose glycation model. Compared to the positive reference compounds rutin and quercetin, having an IC_50_ of 69 ± 0.12 and 104 ± 1.75 μM, respectively, compounds **6**, **7**, **13**, and **17** displayed potent inhibitory activities with an IC_50_ of 37 ± 0.48, 78 ± 3.05, 66 ± 1.89, and 25 ± 0.25 μM, respectively. Compounds **12**, **16**, and **18** showed moderate activity, having an IC_50_ of 260 ± 2.50, 909 ± 5.86, and 265 ± 3.88 μM, respectively. This is the first report to find hypoglycemic compounds from *T. bakis*, unveiling the components responsible for the traditional use of this plant for treating diabetes.

## 3. Materials and Methods

### 3.1. General Experiment Procedures

The Bruker AMS-400 and AMX-500 (Bruker, Billerica, MA, USA) were used to record NMR spectra. For LR-FAB-MS and LR-EI-MS, JEOL MS Route JMS 600H (JEOL Ltd., Akishima, Japan) mass spectrometer was used, and, for HR-FAB-MS, JEOL JMS-HX110 mass spectrometer was used. A UV/visible spectrophotometer was used to accomplish the UV/visible method. The optical rotations were completed on the p-2000 Polarimeter, and the infrared (IR) spectra were recorded on the Attenuated Total Reflectance Infrared Spectrophotometer (ATRIR) FTIR iS50 (Fourier-Transform Infrared Spectrophotometer, Thermo Fisher Scientific, Waltham, MA, USA) in the KBr disc. Electronic Circular Dichorism (ECD) measurements were performed on Jaso-J-8-10 Circular Dichorism Spectro-Polarimeter. Normal silica gel (E. Merck, 70–230 Mesh) was utilized for fractionation by column chromatography. C18 (Wakogel, 38–63 Mesh), Sephadex LH-20 (GE healthcare, Chicago, IL, USA), and normal and reverse-phase HPLC were employed for purification. The purity of the sample was confirmed using normal and reverse-phase precoated TLC. UV light at 254 nm was used to evaluate TLC plates. Dragendroff, ceric sulfate, and vanillin were employed to visualize the spots on TLC plates.

### 3.2. Plant Material

The whole plant of *T. bakis* was collected from the Nuba Mountains in western Sudan from December 2018 to January 2019 and taxonomically identified by Yahya Sulieman Mohamed at Herbarium of Medicinal and Aromatic Plants and Traditional Medicine Research Institute, National Center for Research, Khartoum, Sudan. A voucher specimen (M-95-17-MAPTRI-H) was preserved in the Herbarium of Medicinal and Aromatic Plants and Traditional Medicine Research Institute, National Center for Research, Khartoum, Sudan, for further reference.

### 3.3. Extraction and Isolation

The powdered plant material (5 kg) of *T. bakis* was extracted with 80% EtOH to obtain crude extract (560 g). The extract was suspended in distilled water and partitioned through *n*-hexane (31 g), ethyl acetate (160 g), and *n*-butanol (119 g) in succession, resulting in four fractions. The ethyl acetate (160 g) was subjected to silica gel column chromatography (CC) by using DCM:MeOH (100:0–0:100) as eluent, from which 21 fractions (F_1_–F_21_) were collected. Fr.4 was separated by silica gel CC eluted with hexane/acetone to afford 17 sub-fractions (F_4-1_–F_4-17_), F_4-5_ were further purified by silica gel CC, then compound **21** (7.8 mg) was obtained. Compound **7** (12.3 mg) was purified from F_4-8_ by the same method. F_5_ was subjected to silica gel CC eluted with hexane/acetone solvent system and gave F_5-1_–F_5-14_. Compounds **8** (13.5 mg) and **9** (9.5 mg) were gained from F_5-11_ and F_5-12_, respectively, by repeated silica gel CC. F_6_–F_8_ was found to have a major component, **14** (1.5 g). F_9_ was fractionated by silica gel CC eluted through gradient hexane/acetone to obtain 14 sub-fractions (F_9-1_–F_9-14_). Compounds **12** (8.2 mg), **13** (4.9 mg), and **15** (11.4 mg) were purified from F_9-4_, F_9-3_, and F_9-2_, respectively, by subjecting the semi-pure fraction on silica gel CC again. F_10_, F_13_, F_15_, F_16_, F_17_, and F_18_ were loaded on C18 silica gel CC with gradient elution of 20–100% MeOH for further fractionation. F_10-4_ was subjected to silica gel CC eluted with hexane/acetone solvent system, and then compounds **4** (8.1 mg), **18** (12.1 mg), and **23** (9.0 mg) were purified. F_13-2_ was loaded on silica gel CC by DCM/MeOH elution, resulting in compound **1** (5.2 mg). F_15-3_ was subjected to silica gel CC by gradient DCM/MeOH elution to afford 12 sub-fractions (F_15-3-1_–F_15-3-12_). F_15-3-8_ was further fractionated over silica gel CC with gradient EtOAc/EtOH elution and purified by preparative RP-HPLC, resulting in compounds **5** (19.8 mg) and **6** (15.2 mg), while compound **22** (8.2 mg) was obtained from F_15-9_ by purification on silica gel. Compounds **3** (7.8 mg), **10** (4.8 mg), **16** (14.6 mg), and **17** (12.4 mg) were obtained from F_16-2_ by a similar protocol. Compound **2** (25.8 mg) was isolated from F_17-3_ by subjecting the semi-pure fraction to Sephadex LH-20 with MeOH/H_2_O, followed by purification over silica gel CC using gradient DCM/MeOH/H_2_O elution. Similarly, F_18-5_ was fractionated over silica gel CC eluted with gradient DCM/MeOH/H_2_O to afford 13 sub-fractions. Then, F_18-5-6_, F_18-5-7_, and F_18-5-8_ were separated on Sephadex LH-20 column with MeOH/H_2_O and purified by successive preparative RP-HPLC to yield compounds **11** (15.9 mg), **19** (4.9 mg), and **20** (6.3 mg), respectively. The flow rate during the preparative RP-HPLC was 4 mL/min.

Tinobakisin (**1**): white needle-like crystals; ${\left[\alpha \right]}_{D}^{25}$ −18.18 (c $1.10\times 10^{-3}$, MeOH); UV λ_max_ 244 nm; IR (KBr) ν_max_ 3310, 2943, 2832, 1738, 1589, 1427, 1280, 1073, 1020, 697, 668 cm^−1^; CD [nm (mdeg)]: 217 (5.34); ^1^H-NMR and ^13^C-NMR data, see Table 1; EI-MS [M]^+^, *m*/*z* 390.3; HR-EI-MS [M]^+^ *m*/*z* 390.1687 (calculated for 390.1679, C_21_H_26_O_7_).
Tinobakiside (**10**): amorphous, colorless semisolid; ${\left[\alpha \right]}_{D}^{25}$ −110.16 (c $2.46\times 10^{-3}$, MeOH); UV λ_max_ 248 nm; IR (KBr) ν_max_ 3405, 2918, 1724, 1676, 1506, 1463, 1441, 1389, 1252, 1075, 1021, 602 cm^−1^; CD [nm (mdeg)]: 216 (5.23); ^1^H-NMR and ^13^C-NMR data, see Table 1; FAB-MS [M + H]^+^ *m*/*z* 493.1; HR-FAB-MS [M + H]^+^ *m*/*z* 493.2061 (calculated for 493.2074, C_25_H_33_O_6_).

### 3.4. Acid Hydrolysis

First, 5 mL 1 M HCl was used to dissolve compound **10** (2 mg), and then the mixture was stirred at 80 °C. After 8 h, the reaction mixture was extracted by CH_2_Cl_2_. Then, the obtained aqueous layer was evaporated under a vacuum and diluted multiple times to provide a neutral residue. The residue was subjected to analytical HPLC (Jasco LC-4000, Tokyo, Japan) with a Jasco OR-4090 optical rotation detector. The hydrolyzed sugar moiety indicated a positive rotation [45,46].

### 3.5. ECD Calculation

The ECD calculation of **1** was performed as reported, and the details are provided in Supplementary Data [10,47]. Conformational search with systematic algorithm was performed in Yinfo Cloud Platform (https://cloud.yinfotek.com/, accessed on 26 September 2023) using Confab at MMFF94 force field. Conformers were filtered by an RMSD threshold of 0.5 Å and an energy window of 7 kcal/mol. The energies and populations of dominative conformers are provided in Table S1. Structures for ECD calculation are shown in Table S2. All structures were confirmed by vibration frequency analysis, and no imaginary frequencies were found. Table S3 indicates the standard orientations of all configurations for ECD calculation at B3LYP/6-311G(d,p) level in methanol.

### 3.6. Anti-Glycation Activity

The anti-glycation effect was performed by the BSA (bovine serum albumin)–glucose glycation model in vitro, as previously described [48]. Glucose (0.5 M), BSA (10 mg/mL), and sodium azide (NaN_3_) (0.1 mM) were dissolved in sodium phosphate buffer (pH 7.4) as the reaction reagent to perform with or without the tested sample. The test compounds and standard references (rutin and quercetin) were dissolved in 10% DMSO. BSA without a glycating agent was used as a negative control. All samples were initially screened at 1 mM concentration. The assay was performed in triplicates, and a final reaction volume of 200 μL was maintained in each well of a 96-well plate. The reaction mixture was incubated at 37 °C for 7 days in a dark, sterile condition. The fluorescence (excitation 340 nm and emission 440 nm) was measured using a Varioskan Lux microtitre plate reader (Thermo Fisher Scientific, Waltham, MA, USA).

The percentage (%) of inhibition was calculated by using the following formula:

Inhibition (%) of fluorescence = (1 − Fluorescence of test compound/glycated BSA) × 100.

The compounds that exhibited more than 50% inhibition were processed for IC_50_. The two-fold dilution was made, and multiple concentrations (1, 0.5, 0.25, 0.125, 0.06, and 0.03 mM) of active compounds and standards (rutin and quercetin) were incubated with BSA-glucose at 37 °C for 7 days. The fluorescence was measured, and then the IC_50_ (μM) was calculated using the EZ-FIT Enzyme Kinetics Program (Perrella Scientific Inc., Amherst, MA, USA).

## 4. Conclusions

In summary, our phytochemical investigation of *Tinospora bakis* uncovered a plethora of clerodane furanoditerpenoids, including the discovery of two new compounds, tinobakisin (**1**) and tinobakiside (**10**), and provided clarifications on previously reported compounds. The observed superior anti-glycation effects of compounds **6**, **7**, **13**, and **17** underscore the potential therapeutic relevance of *T. bakis* in managing diabetic complications. This research offers the first in-depth chemical profile of *T. bakis*. It lays the foundation for future studies to understand the mechanistic details of these compounds’ biological activities and their potential therapeutic applications, thus paving the way for new avenues in treating chronic diseases. More in vivo pharmacological and mechanism investigation might be required to further understand the active components’ antidiabetic properties, leading to the discovery of potential lead compounds for drug discovery.

## Figures and Tables

**Table 1:** NMR data of compounds **1** and **10.**

No.	1 *^a^*		10 *^b^*	
	*δ*_H_	*δ*_C_, Multipilicity	*δ*_H_	*δ*_C_, Multipilicity
1	2.27 overlapped 2.04 overlapped	30.1, CH_2_	2.90 ddd (20.0, 6.5, 2.5) 2.54 dd (20.0, 6.5)	22.5, CH_2_
2	4.46 m	65.1, CH	6.30 dd (6.5, 2.5)	124.3, CH
3	6.57 d (3.2)	140.4, CH	-	149.2, C
4	-	141.1, C	-	200.5, C
5	-	36.0, C	-	46.8, C
6	2.03 overlapped 1.75 m	30.6, CH_2_	2.28 overlapped 1.06 td (14.0, 4.0)	30.9, CH_2_
7	2.10 overlapped 1.64 ddd (14.0, 10.0, 2.8)	39.6, CH_2_	2.16 dq (14.0, 4.0) 1.72 tt (14.0, 4.0)	20.3, CH_2_
8	-	90.4, C	2.47 br t (4.0)	50.1, CH
9	-	50.0, C	-	37.6, C
10	1.92 d (5.6)	47.0, CH	2.29 overlapped	45.3, CH
11	2.27 overlapped 2.05 overlapped	47.0, CH_2_	2.37 dd (15.0, 4.0) 1.80 dd (15.0, 12.5)	41.3, CH_2_
12	5.15 dd (11.2, 4.8)	74.3, CH	5.57 dd (12.5, 4.0)	72.3, CH
13	-	127.6, C	-	126.8, C
14	6.73 d (1.2)	110.9, CH	6.54 d (2.0)	109.6, CH
15	7.39 t (1.2)	144.1, CH	7.49 t (2.0)	145.0, CH
16	7.54 brs	142.0, CH	7.59 br s	141.4, CH
17	-	178.3, C	-	174.8, C
18	-	169.5		
19	1.44 s	30.6, CH_3_	1.28 s	29.0, CH_3_
20	1.05 s	22.2, CH_3_	0.97 s	26.6, CH_3_
1′	-		4.62 d (7.0)	102.3, CH
2′	-		3.38 overlapped	74.6, CH
3′	-		3.32 overlapped	78.3, CH
4′	-		3.34 overlapped	71.3, CH
5′	-		3.39 overlapped	77.6, CH
6′	-		3.84 dd (12.0, 1.8) 3.65 dd (12.0, 5.2)	62.5, CH_2_
OCH_3_	3.73 s	52.1, CH_3_		

*^a^* ^1^H-NMR data (*δ*) were measured in CD_3_OD at 400 MHz. ^13^C-NMR data (*δ*) were measured in CD_3_OD at 125 MHz. *^b^* ^1^H-NMR data (*δ*) were measured in CD_3_OD at 500 MHz. ^13^C-NMR data (*δ*) were measured in CD_3_OD at 125 MHz.

## Data Availability

The data presented in this study are available in article and Supplementary Materials.

## Supplementary Materials

The following supporting information can be downloaded at https://www.mdpi.com/article/10.3390/molecules29010154/s1. Figure S1. UV spectrum of compound **1**. Figure S2. IR spectrum of compound **1**. Figure S3. EI-MS spectrum of compound **1**. Figure S4. HR-EI-MS spectrum of compound **1**. Figure S5. CD spectrum of compound **1**. Figure S6. ^1^H-NMR spectrum of compound **1** (CD_3_OD, 400 MHz). Figure S7. ^13^C-NMR spectrum of compound **1** (CD_3_OD, 125 MHz). Figure S8. DEPT spectrum of compound **1**. Figure S9. COSY spectrum of compound **1**. Figure S10. HSQC spectrum of compound **1**. Figure S11. HMBC spectrum of compound **1**. Figure S12. NOESY spectrum of compound **1**. Figure S13. IR spectrum of compound **10**. Figure S14. IR spectrum of compound **10**. Figure S15. CD spectrum of compound **10**. Figure S16. FAB-MS spectrum of compound **10.** Figure S17. HR-FAB-MS spectrum of compound **10**. Figure S18. ^1^H-NMR spectrum of compound **10** (CD_3_OD, 500 MHz). Figure S19. ^13^C-NMR spectrum of compound **10** (CD_3_OD, 125 MHz). Figure S20. DEPT spectrum of compound **10**. Figure S21. COSY spectrum of compound **10**. Figure S22. HSQC spectrum of compound **10**. Figure S23. HMBC spectrum of compound **10**. Figure S24. NOESY spectrum of compound **10**. S1. Detailed methodology of ECD calculation of compound **1**. Table S1. Energies of all Conformers of compound **1** at MMFF94 force field. Table S2. Energies of all configurations of compound **1** at B3LYP/6-311G(d,p) in methanol. Table S3. Standard orientations of all configurations of compound **1** at B3LYP/6-311G(d,p) level in Methanol.
