# Supplementary material for: Clerodane Furanoditerpenoids from Tinospora bakis (A.Rich.) Miers (Menispermaceae)
Source: Molecules. 2023 Dec 26;29(1):154. doi: 10.3390/molecules29010154 (PMC10780120; doi:10.3390/molecules29010154)
Supplement: Supplementary file 1 [file molecules-29-00154-s001.zip › molecules-2768773-supplementary.pdf]

Supplementary data

Clerodane furanoditerpenoids from *Tinospora bakis* (A.Rich.) Miers (Menispermaceae)

Ahmed Saeed Kabbashi [1,2,†], Maazah Abdul Sattar [1,†], Muhammad Aamer[1], Nimra Naz Siddiqui [3], Muhammad Kamran [1], Aneela Fayaz [1], Humera Jahan [3], Farooq Ahmad Khan [1,4,*] and Yan Wang [1,*]

1 H. E. J. Research Institute of Chemistry, International Center for Chemical and Biological Sciences, University of Karachi, Karachi 75270, Pakistan; ahmedsak88@gmail.com (A.S.K.); maazahmughal@gmail.com (M.A.S.); m.aamer00786@gmail.com (M.A.); mkchemist785@gmail.com (M.K.); aneelafayaz1993@gmail.com (A.F.).

2 Department of Microbiology, Faculty of Pure and Applied Sciences, International University of Africa, Khartoum, Sudan

3 Dr. Panjwani Center for Molecular Medicine and Drug Research, International Center for Chemical and Biological Sciences, University of Karachi, Karachi-75270, Pakistan; nazsiddiqui92@gmail.com (N.N.S.), jahan_pcmd@yahoo.com (H.J.)

4 Third World Center (TWC) for Chemical Sciences, International Center for Chemical & Biological Sciences, University of Karachi, Karachi-75270, Pakistan

* Correspondence: yan.wang@iccs.edu (Y.W.); farooq.khan@iccs.edu (F.-A.K.)

† These authors contributed equally to this work.

List of Contents

Figure S1. UV spectrum of compound **1** 3
Figure S2. IR spectrum of compound **1** 4
Figure S3. EI-MS spectrum of compound **1** 4
Figure S4. HR-EI-MS spectrum of compound **1** 5
Figure S5. CD spectrum of compound **1** 5
Figure S6. ^{1}H NMR spectrum of compound **1** (CD_3OD, 400 MHz) 6
Figure S7. ^{13}C NMR spectrum of compound **1** (CD_3OD, 125 MHz) 6
Figure S8. DEPT spectrum of compound **1** 7
Figure S9. ^{1}H-^{1}H COSY spectrum of compound **1** 7
Figure S10. HSQC spectrum of compound **1** 8
Figure S11. HMBC spectrum of compound **1** 8
Figure S12. NOESY spectrum of compound **1** 9
Figure S13. UV spectrum of compound **10** 10
Figure S14. IR spectrum of compound **10** 11
Figure S15. CD spectrum of compound **10** 12
Figure S16. FAB-MS spectrum of compound **10** 13
Figure S17. HR-FAB-MS spectrum of compound **10** 13
Figure S18. ^{1}H NMR spectrum of compound **10** (CD_3OD, 500 MHz) 14
Figure S19. ^{13}C NMR spectrum of compound **10** (CD_3OD, 125 MHz) 14
Figure S20. DEPT spectrum of compound **10** 15
Figure S21. ^{1}H-^{1}H COSY spectrum of compound **10** 15
Figure S22. HSQC spectrum of compound **10** 16
Figure S23. HMBC spectrum of compound **10** 16
Figure S24. NOESY spectrum of compound **10** 17
S1. ECD Calculation of compound **1** 17
S1.1 Energies at MMFF94 force field 17
S1.2 Energies at B3LYP theory level 17
S1.3 Coordinates at B3LYP theory level 19

THERMO ELECTRON ~ VISIONpro SOFTWARE V4.10

Operator Name	STUDENT	Date of Report	7/11/2023
Department	Analytical Lab Nanotechnology center	Time of Report	4:27:54PM
Organization	ICCBS,Karachi University		
Information	MAAZAH/DR .YAN		

Scan Graph

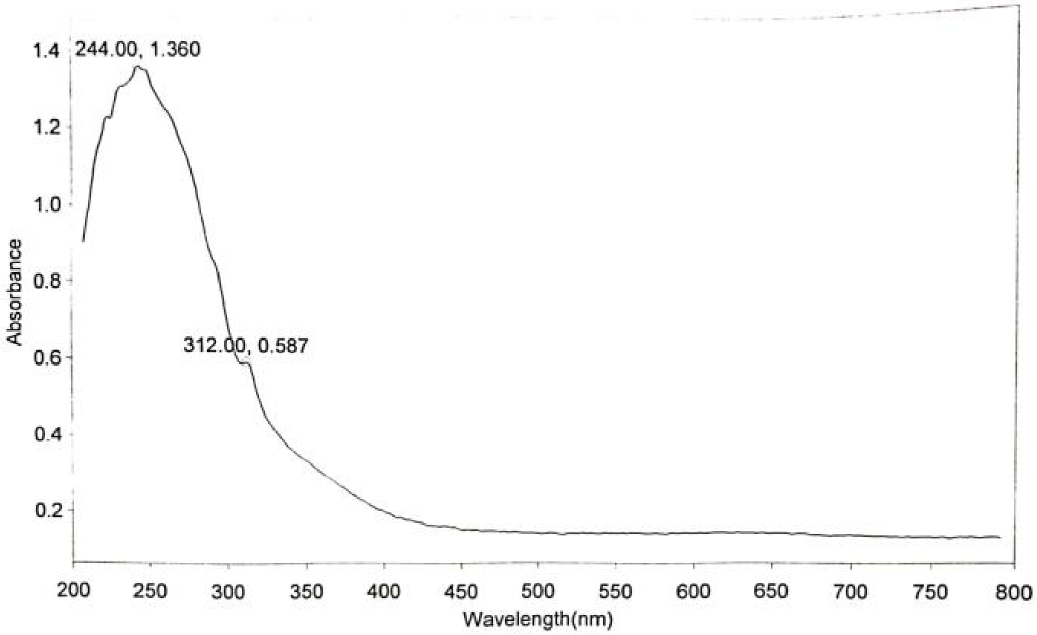

Results Table - AS13-2-6.sre,AS-13-2-6,Cycle01

nm	A	**Peak Pick Method**
244.00	1.360	Find 8 Peaks Above -3.0000 A
312.00	0.587	Start Wavelength200.00 nm
		Stop Wavelength600.00 nm
		Sort By Wavelength
Sensitivity	Medium	

Figure S1. UV spectrum of compound **1**

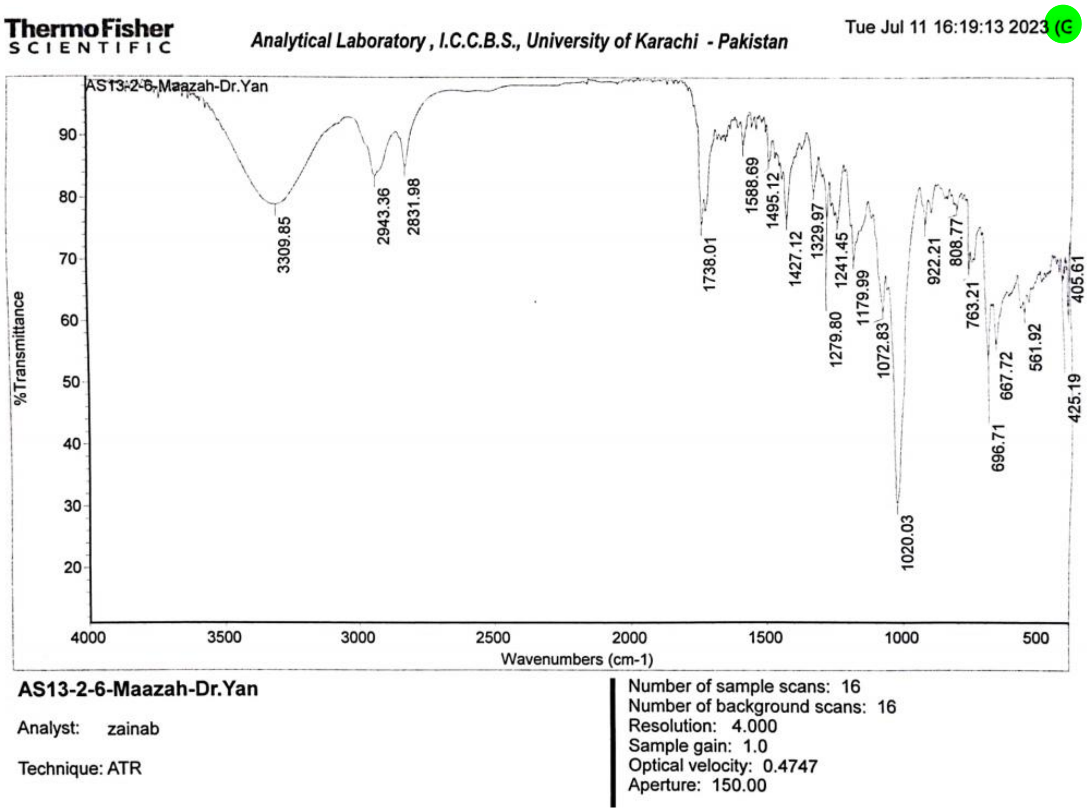

Figure S2. IR spectrum of compound **1**

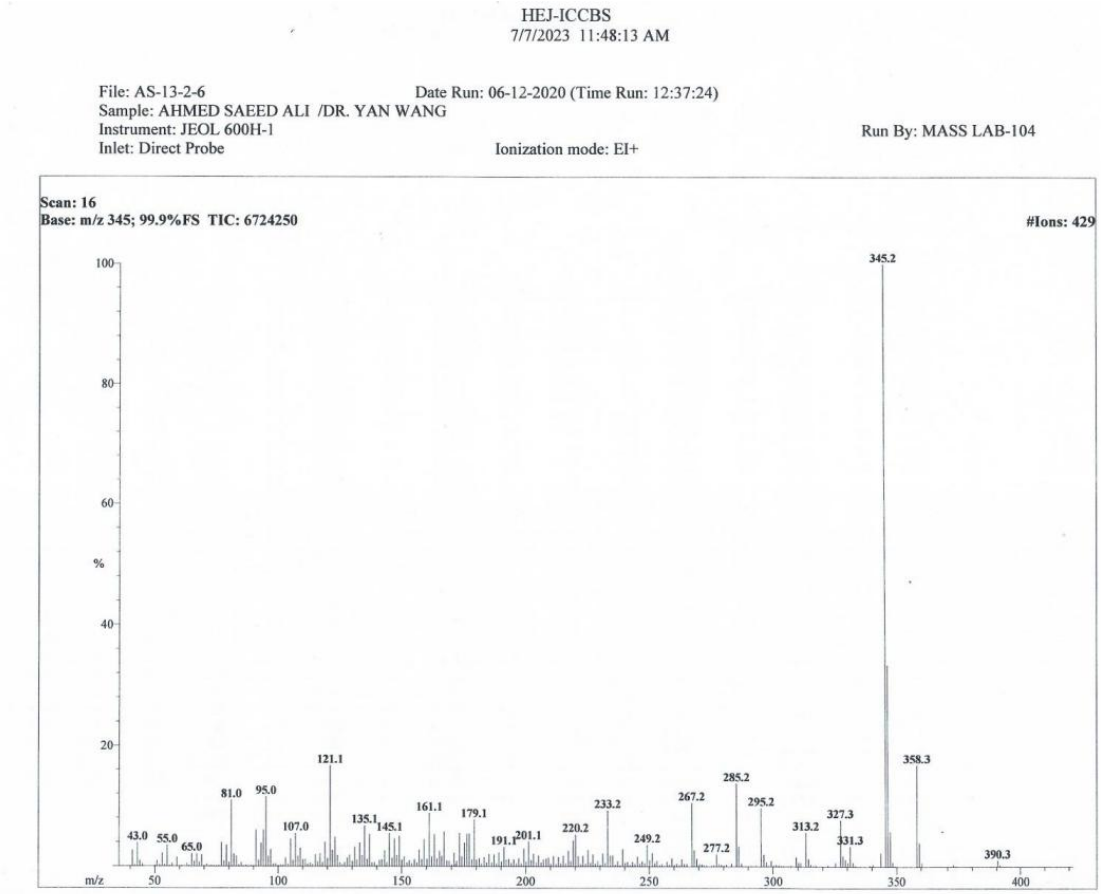

Figure S3. EI-MS spectrum of compound **1**

Mass	Relative Intensity	Theoretical Mass	Delta [ppm]	Delta [mmu]	RDB	Composition
		327.1444	32.4	10.6	5.5	$C_{16}H_{23}O_7$
345.1727	38.3	345.1702	7.2	2.5	8.5	$C_{20}H_{25}O_5$
		345.1643	24.2	8.4	17.5	$C_{27}H_{21}$
		345.1855	-37.0	-12.8	12.5	$C_{24}H_{25}O_2$
346.1774	8.1	346.1780	-1.9	-0.7	8.0	$C_{20}H_{26}O_5$
		346.1722	15.1	5.2	17.0	$C_{27}H_{22}$
347.1822	1.3	347.1800	6.5	2.3	16.5	$C_{27}H_{23}$
		347.1858	-10.4	-3.6	7.5	$C_{20}H_{27}O_5$
		347.1706	33.6	11.7	3.5	$C_{16}H_{27}O_8$
358.1418	3.9	358.1416	0.5	0.2	10.0	$C_{20}H_{22}O_6$
		358.1358	16.9	6.0	19.0	$C_{27}H_{18}O_1$
359.1568	1.0	359.1495	20.3	7.3	9.5	$C_{20}H_{23}O_6$
		359.1647	-22.1	-8.0	13.5	$C_{24}H_{23}O_3$
		359.1436	36.7	13.2	18.5	$C_{27}H_{19}O_1$
368.9932	1.7	368.9977	-12.0	-4.4	28.5	$C_{28}H_1O_2$
		368.9883	13.5	5.0	15.5	$C_{17}H_5O_{10}$
		369.0035	-27.9	-10.3	19.5	$C_{21}H_5O_7$
390.1687	2.0	390.1679	2.3	0.9	9.0	$C_{21}H_{26}O_7$
		390.1620	17.3	6.8	18.0	$C_{28}H_{22}O_2$
		390.1831	-36.8	-14.4	13.0	$C_{25}H_{26}O_4$

Figure S4. HR-EI-MS spectrum of compound **1**

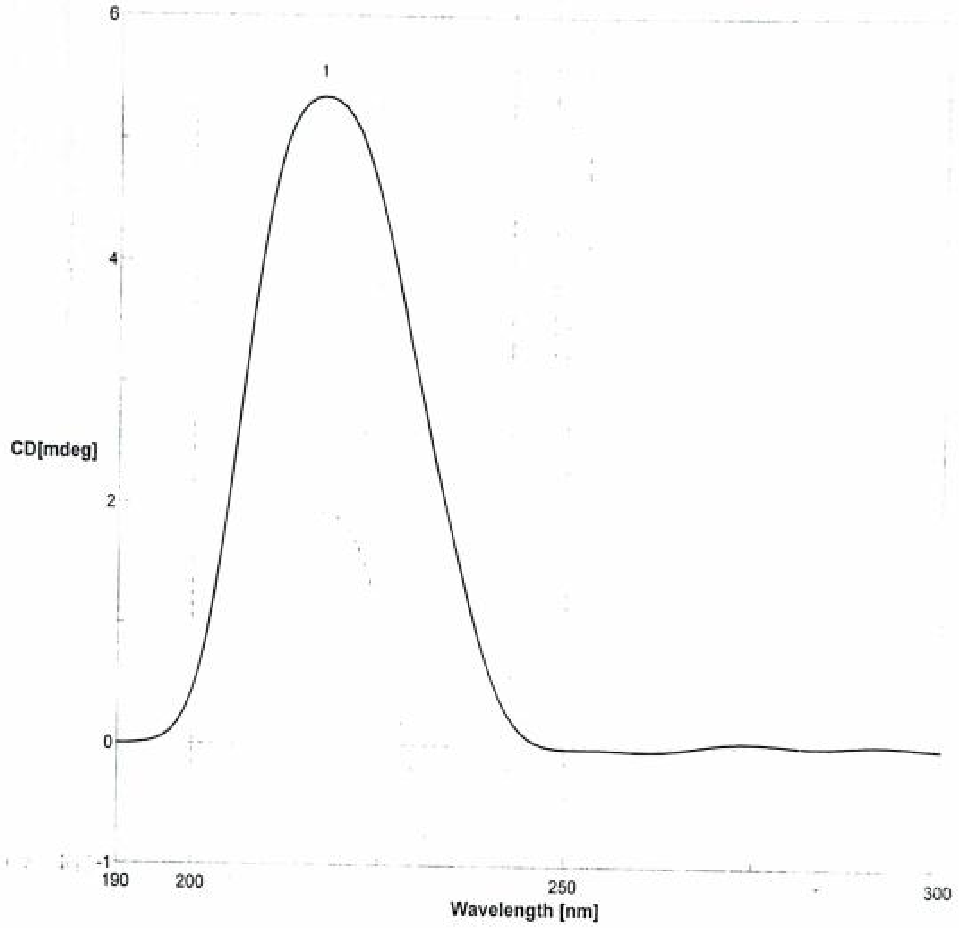

Date/Time	4/2/2012 4:51AM
Operator	Zubair Ahmed
File Name	Memory#6
Sample Name	AS13-2-6
Comment	A16-2-4-3-30-2/Maazah/Dr.Yan

No.	nm	CD[mdeg]
1	217	5.34489

Figure S5. CD spectrum of compound **1**

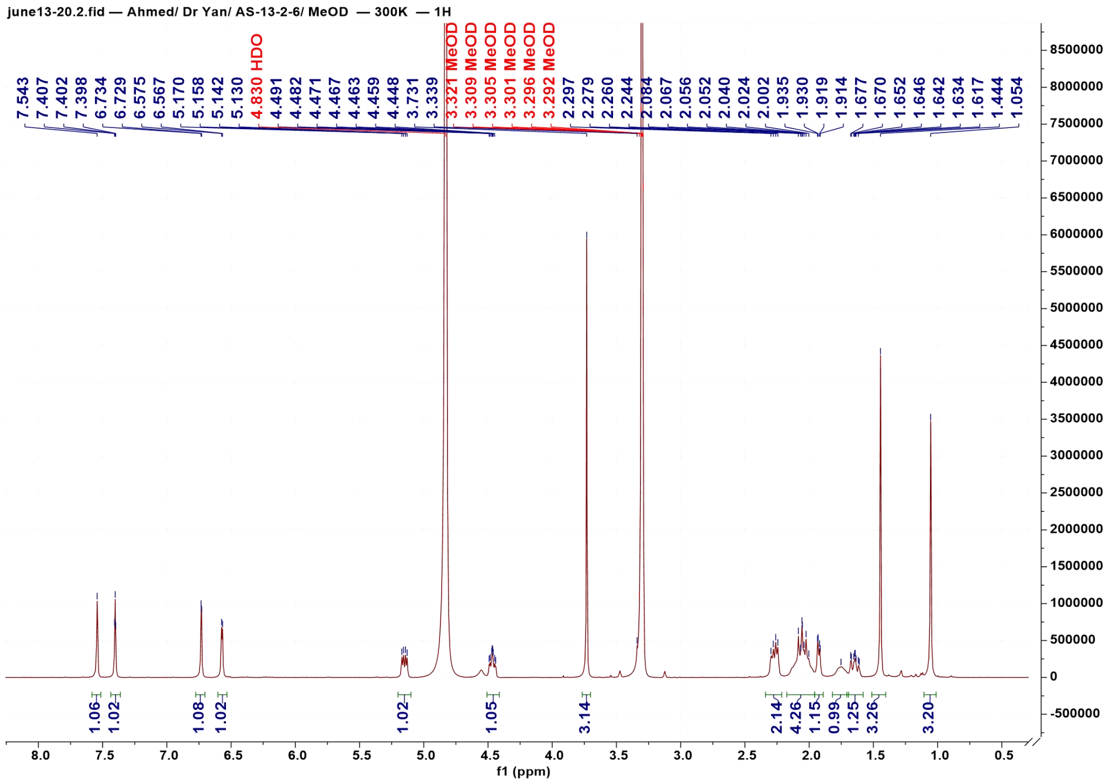

Figure S6. ^{1}H NMR spectrum of compound **1** (CD_3OD, 400 MHz)

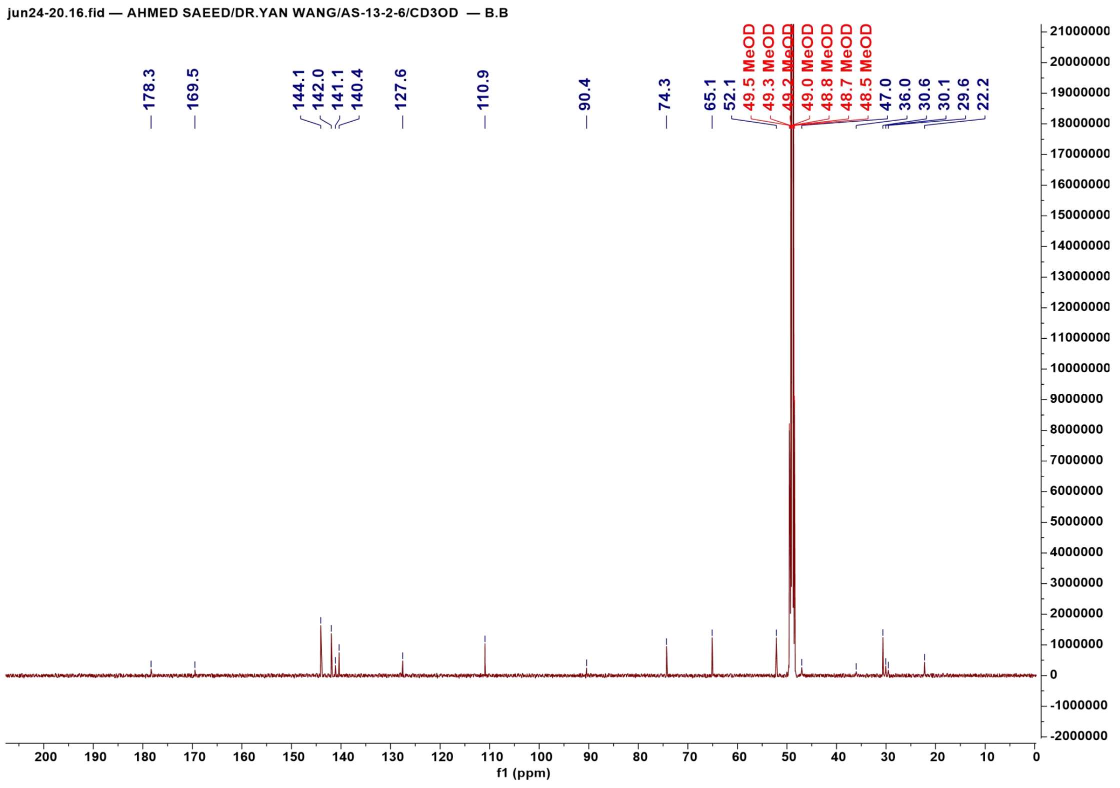

Figure S7. ^{13}C NMR spectrum of compound **1** (CD_3OD, 125 MHz)

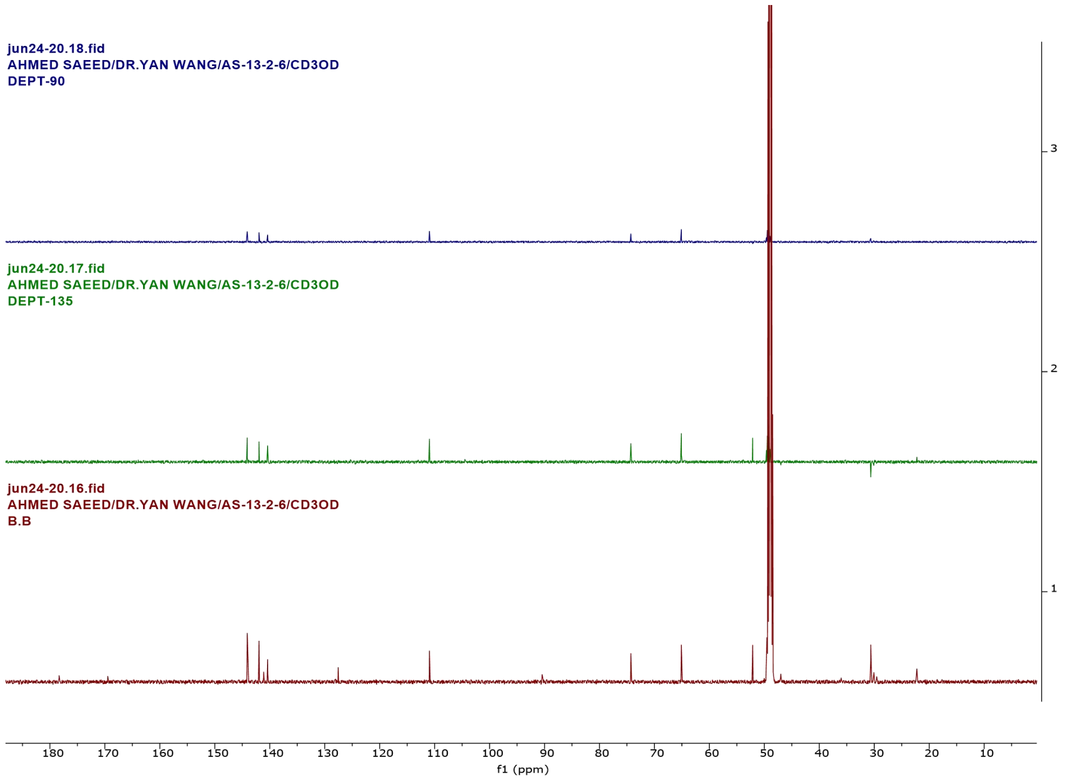

Figure S8. DEPT spectrum of compound **1**

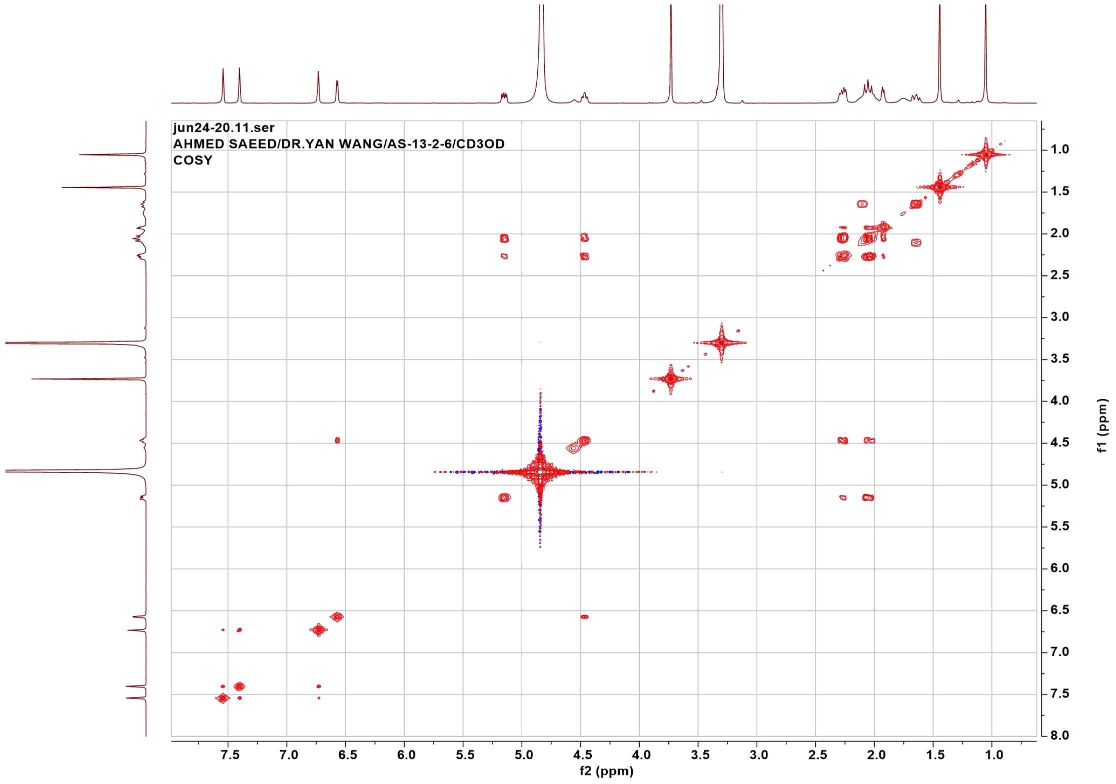

Figure S9. ^{1}H-^{1}H COSY spectrum of compound **1**

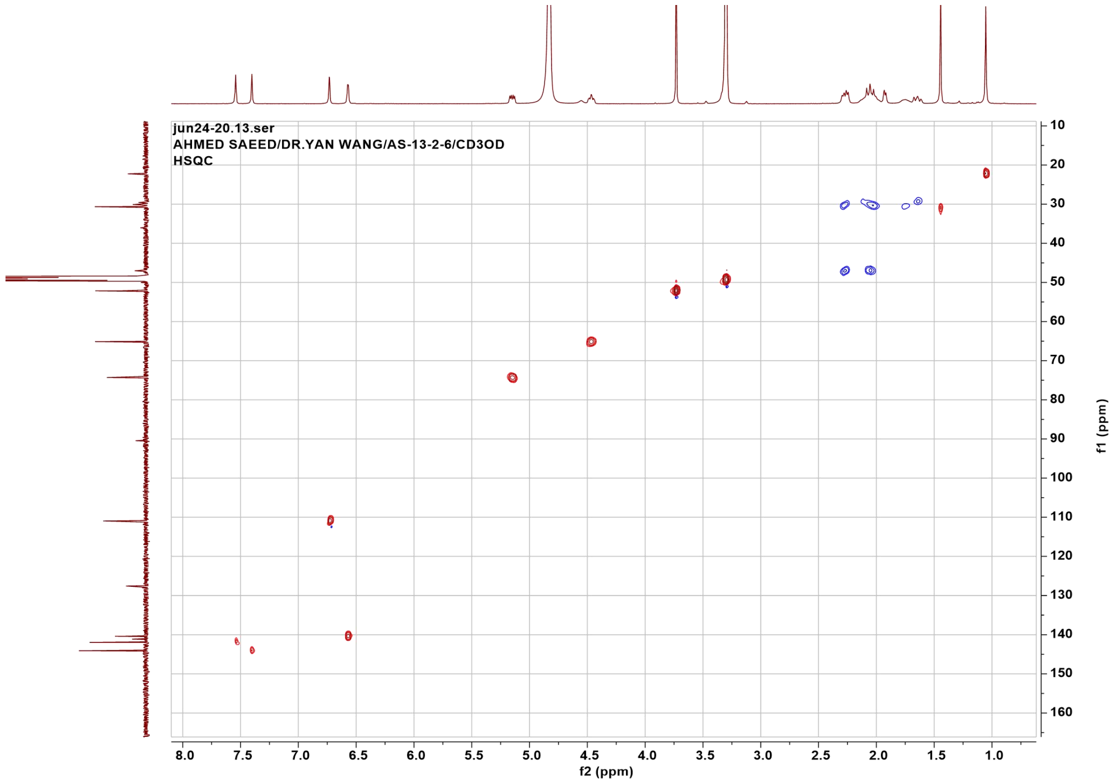

Figure S10. HSQC spectrum of compound **1**

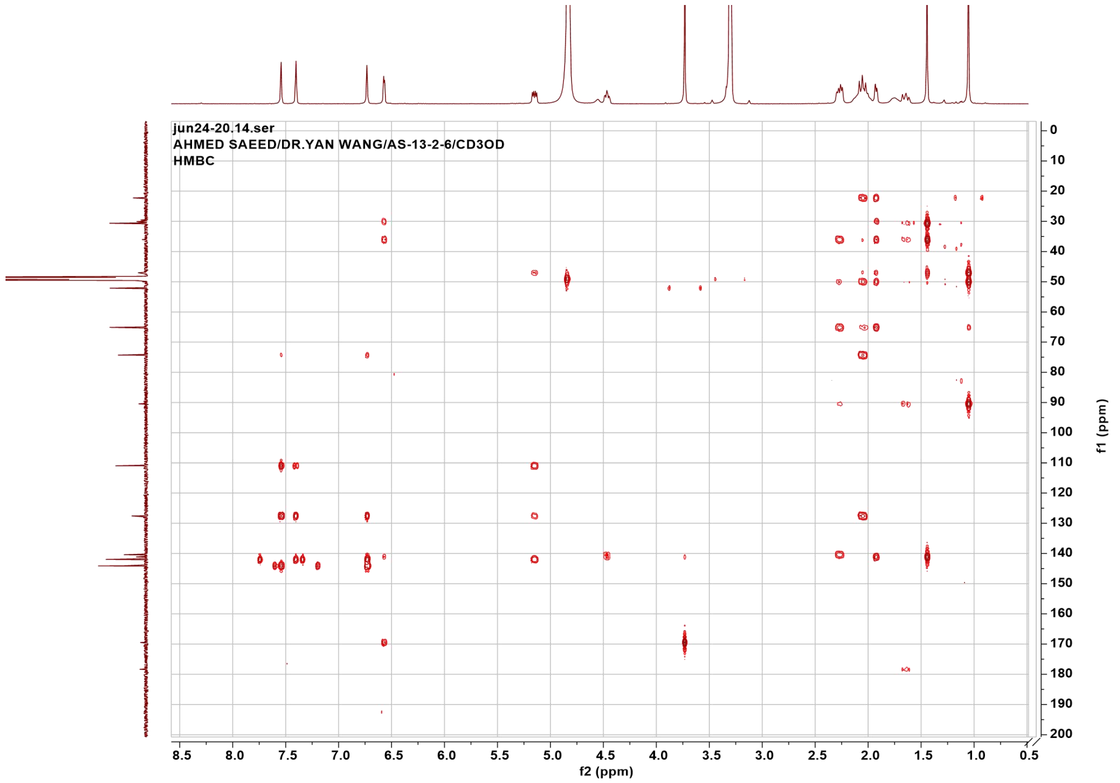

Figure S11. HMBC spectrum of compound **1**

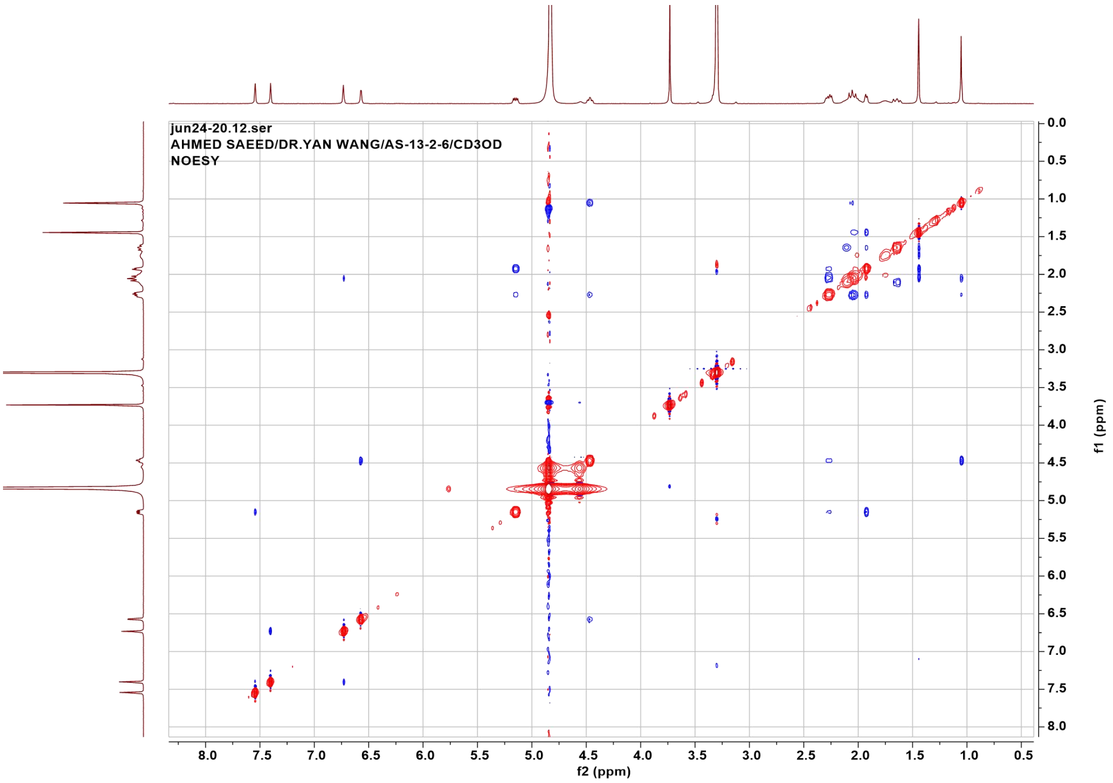

Figure S12. NOESY spectrum of compound **1**

THERMO ELECTRON ~ VISIONpro SOFTWARE V4.10

Operator Name	STUDENT	Date of Report	7/11/2023
Department	Analytical Lab Nanotechnology center	Time of Report	4:28:31PM
Organization	ICCBS,Karachi University		
Information	MAAZAH/DR .YAN		

Scan Graph

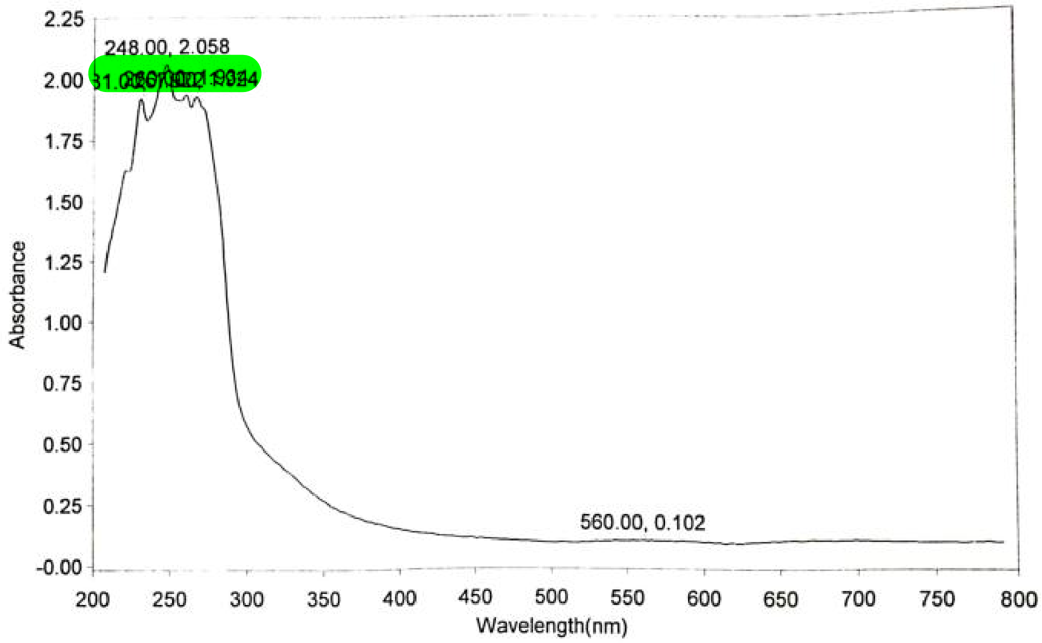

Results Table - AS16-1-2-4-3-30-20.sre,AS16-2-4-3-30-20,Cycle01

nm	A	Peak Pick Method	
231.00	1.922	Find 8 Peaks Above -3.0000 A	
248.00	2.058	Start Wavelength200.00 nm	
260.00	1.934	Stop Wavelength600.00 nm	
267.00	1.924	Sort By Wavelength	
560.00	0.102	Sensitivity	Medium

Figure S13. UV spectrum of compound **10**

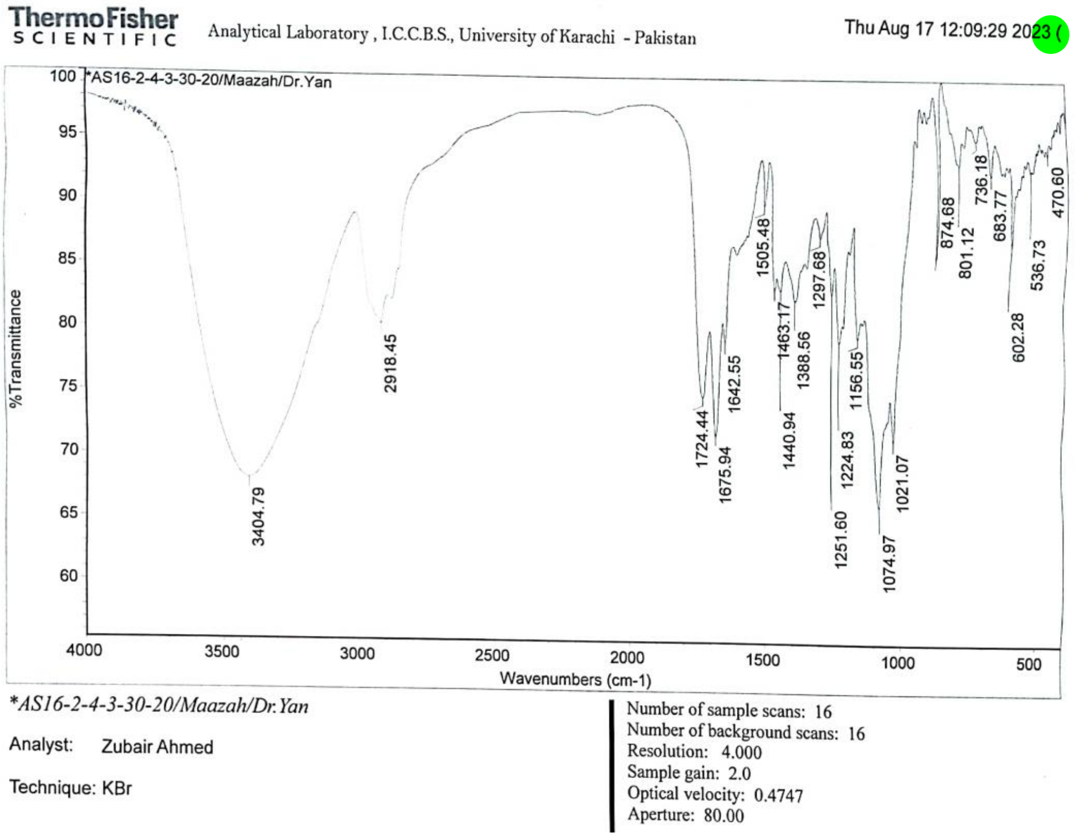

Figure S14. IR spectrum of compound **10**

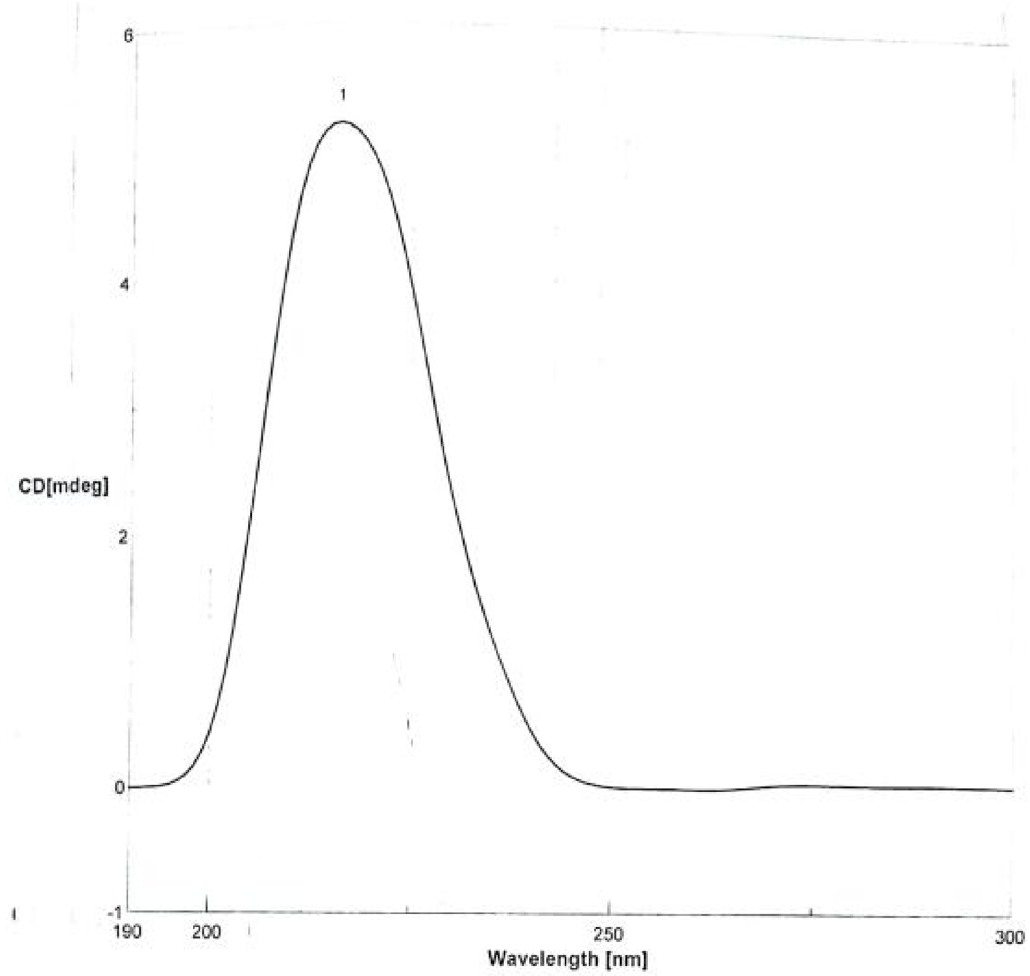

Date/Time	4/2/2012 4:40AM
Operator	Salar
File Name	Memory#4
Sample Name	A16-2-4-3-30-20
Comment	A16-2-4-3-30-20/Maazah/Dr.Yan

No.	nm	CD[mdeg]
1	216	5.22683

Figure S15. CD spectrum of compound **10**

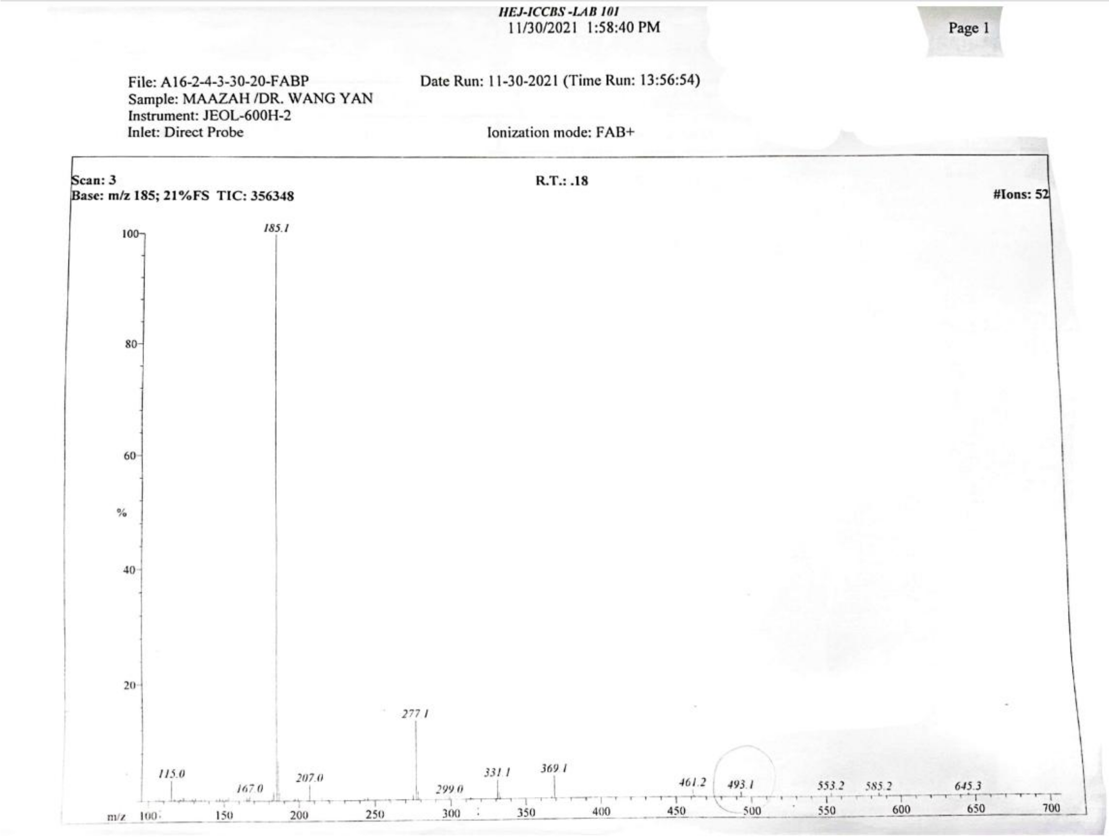

Figure S16. FAB-MS spectrum of compound **10**

HEJ-MASS LAB -ICCBS		JEOL HX 110 MASS SPECTROMETER (FAB-HR)		
STUDENT NAME	MAAZAH	SAMPLE CODE	DATE	6/10/2022
SUPERVISOR NAME	DR. YAN	A16-2-4-3-30-20	FAB (+VE / -VE)	+VE

Measured	Theoretical Mass	Delta [ppm]	Delta [mmu]	RDB	Composition
493.2061	493.2074	-2.6	-1.3	9.5	**C25 H33 O10**
	493.2015	9.3	4.6	18.5	C32 H29 O5
	493.1956	21.2	10.5	27.5	C39 H25
	493.2168	-21.6	-10.7	22.5	C36 H29 O2

Figure S17. HR-FAB-MS spectrum of compound **10**

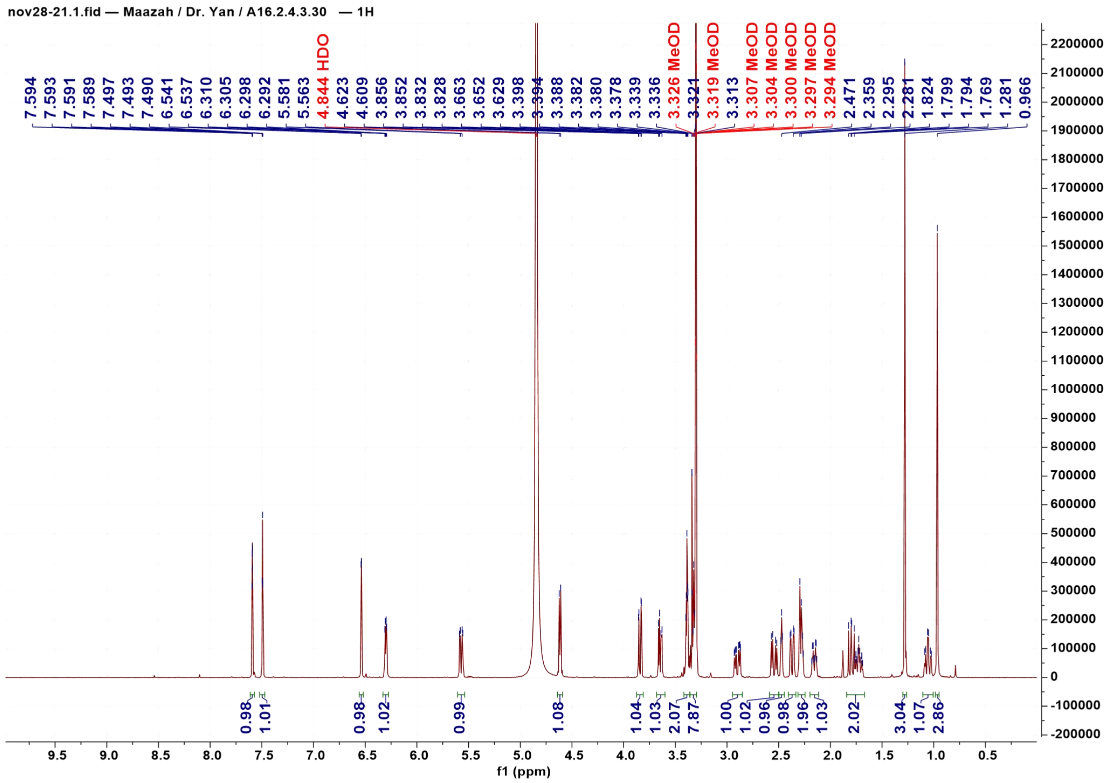

Figure S18. ^{1}H NMR spectrum of compound **10** (CD_3OD, 500 MHz)

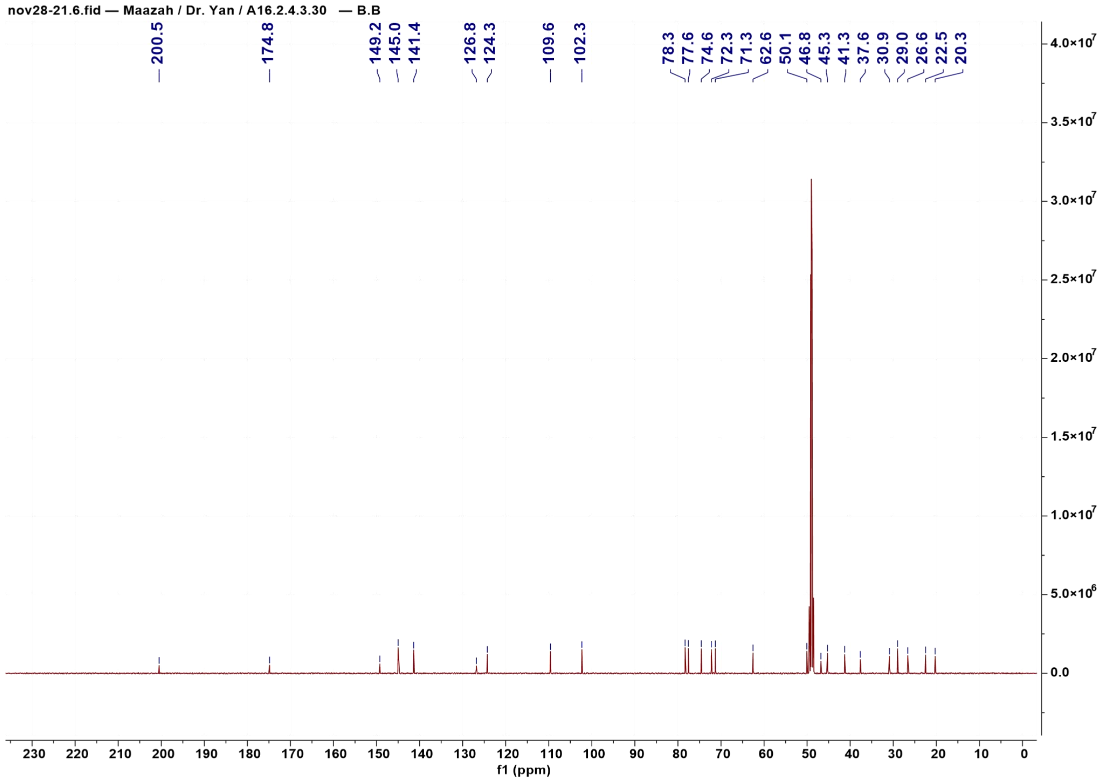

Figure S19. ^{13}C NMR spectrum of compound **10** (CD_3OD, 125 MHz)

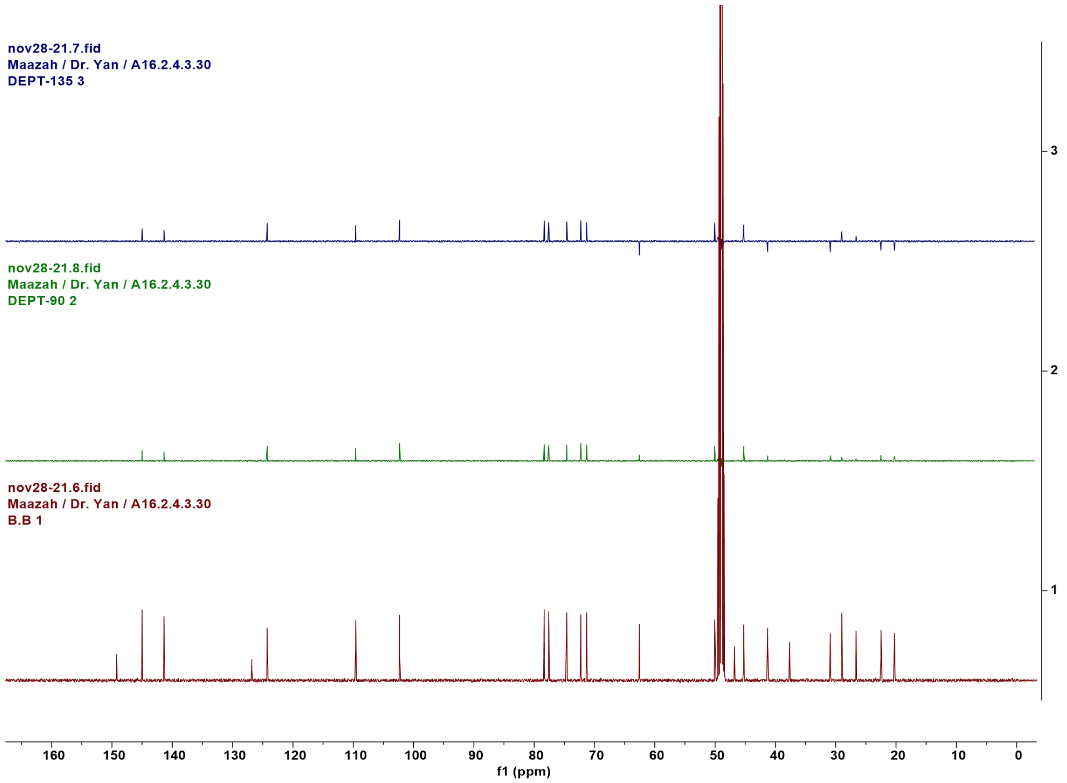

Figure S20. DEPT spectrum of compound **10**

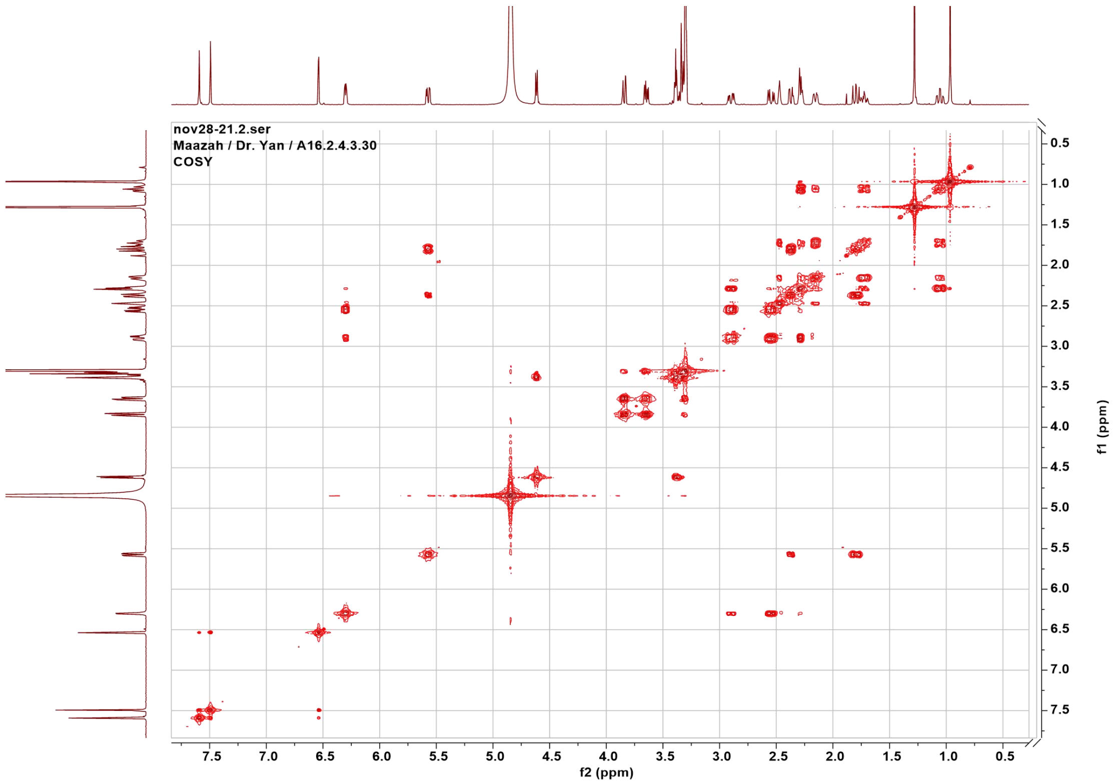

Figure S21. ^{1}H-^{1}H COSY spectrum of compound **10**

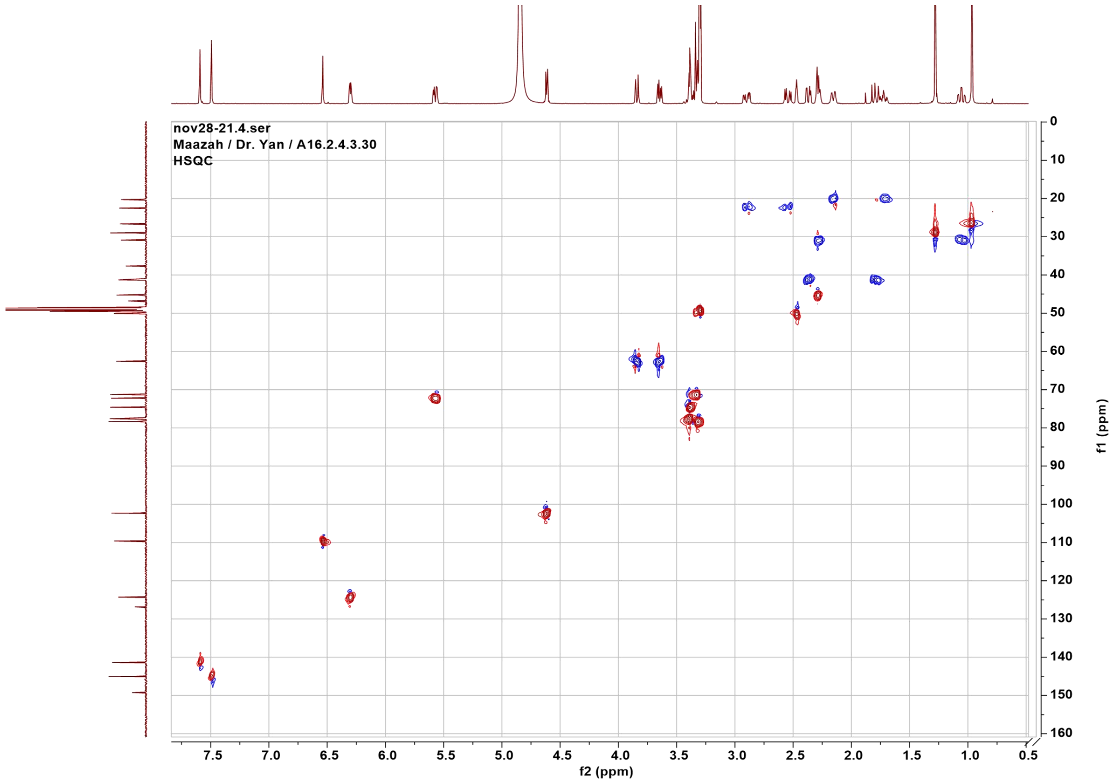

Figure S22. HSQC spectrum of compound **10**

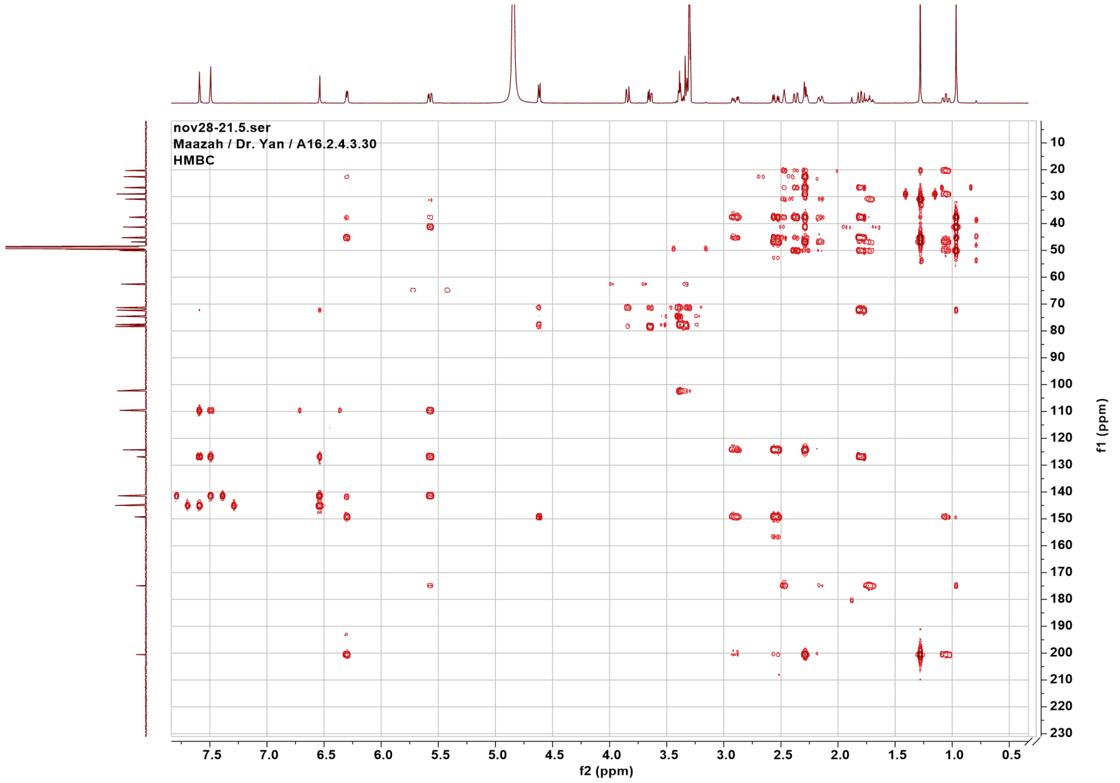

Figure S23. HMBC spectrum of compound **10**

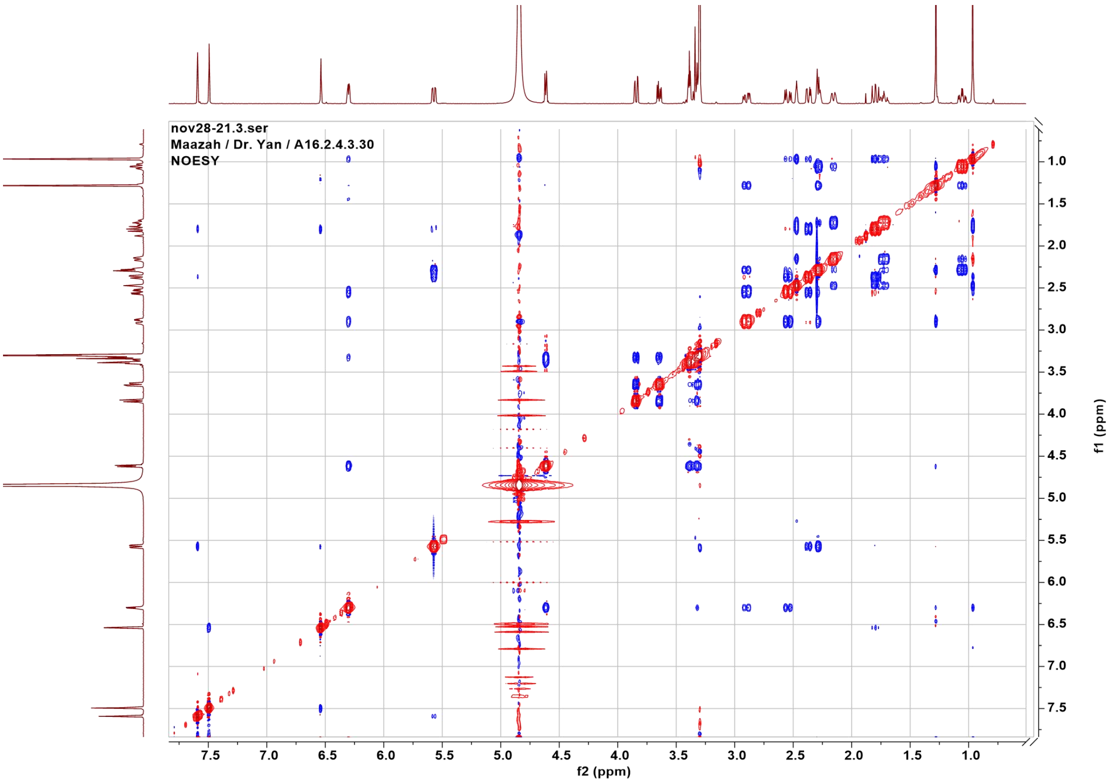

Figure S24. NOESY spectrum of compound **10**

S1. ECD Calculation of compound 1

S1.1 Energies at MMFF94 force field

Conformational search with systematic algorithm was performed in Yinfo Cloud Platform (https://cloud.yinfotek.com/) using Confab at MMFF94 force field. Conformers were filtered by RMSD threshold of 0.5 Å and energy window of 7 kcal/mol. The energies and populations of dominative conformers were provided in **Table S1**.

Table S1. Energies of all Conformers of compound **1** at MMFF94 force field.

Conformer	Energy (kcal/mol)	Population (%)
1	84.31	61.9
2	84.73	30.4
3	85.68	6.1
4	86.49	1.6

S1.2 Energies at B3LYP theory level

Structures for ECD calculation were shown in **Table S2**. All structures were confirmed by vibration frequency analysis that no imaginary frequencies were found.

Table S2. Energies of all configurations of compound **1** at B3LYP/6-311G(d,p) in

methanol.

Conformation	Structure	E (Hartree)	E (kcal/mol)	Population (%)
2	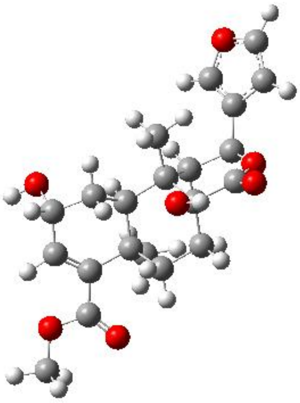	-1342.779924	-842607.12	59.66
1	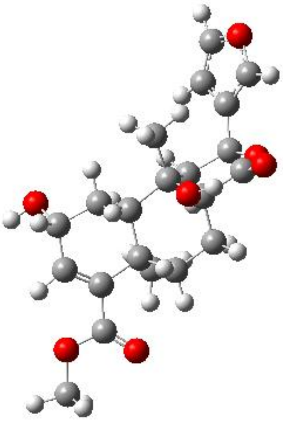	-1342.779004	-842606.54	22.53
4	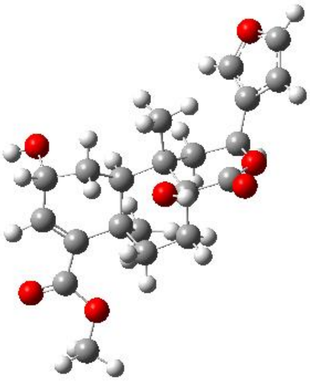	-1342.778486	-842606.21	13.01
3	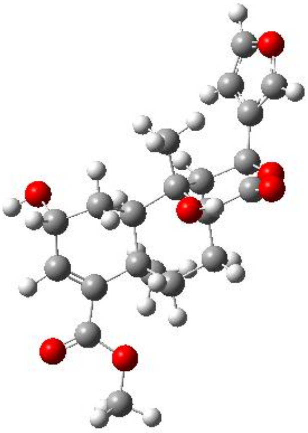	-1342.777544	-842605.62	4.8

S1.3 Coordinates at B3LYP theory level

Table S3. Standard orientations of all configurations of compound **1** at B3LYP/6-311G(d,p) level in Methanol.

Conformer 1

Center	Atomic	Atomic	Coordinates (Angstroms)		
Number	Number	Type	X	Y	Z
1	6	0	-2.395217	2.101363	1.538871
2	6	0	-3.443155	1.356569	0.766458
3	6	0	-3.169388	0.324963	-0.048312
4	6	0	-1.747356	-0.188620	-0.372610
5	6	0	-0.650412	0.756186	0.284936
6	6	0	-1.126140	1.276936	1.658802
7	6	0	-1.597903	-1.638081	0.173474
8	6	0	-0.173749	-2.188441	0.062015
9	6	0	0.819794	-1.307823	0.858537
10	6	0	0.820462	0.163908	0.361670
11	6	0	2.210050	-1.950045	0.743728
12	8	0	2.985972	-1.697571	-0.322673
13	6	0	2.842352	-0.518188	-1.169965
14	6	0	1.473650	0.183138	-1.047444
15	6	0	4.061181	0.344226	-0.947641
16	6	0	4.234130	1.659540	-1.269255
17	8	0	5.508238	2.059247	-0.997531
18	6	0	6.161900	0.974266	-0.492751
19	6	0	5.334385	-0.102473	-0.444950
20	6	0	1.709704	1.005090	1.306915
21	6	0	-1.650444	-0.180468	-1.927468
22	8	0	2.574934	-2.765658	1.567187
23	6	0	-4.340334	-0.390598	-0.665717
24	8	0	-4.317134	-1.505668	-1.154157
25	8	0	0.460788	-1.350469	2.234355
26	8	0	-2.049727	3.343546	0.898081
27	8	0	-5.483672	0.338114	-0.619274
28	6	0	-6.648660	-0.319395	-1.139628
29	1	0	-2.783845	2.313599	2.548136
30	1	0	-4.476807	1.665315	0.896871
31	1	0	-0.577833	1.644948	-0.356507
32	1	0	-1.294764	0.448974	2.354297
33	1	0	-0.371074	1.923505	2.106985
34	1	0	-2.291004	-2.294432	-0.356212
35	1	0	-1.883686	-1.654923	1.230687

36	1	0	0.139375	-2.282754	-0.984726
37	1	0	-0.137005	-3.197836	0.488131
38	1	0	2.912304	-0.941293	-2.178661
39	1	0	1.590098	1.224865	-1.366746
40	1	0	0.813146	-0.278710	-1.775033
41	1	0	3.590405	2.419071	-1.685468
42	1	0	7.192417	1.135866	-0.215688
43	1	0	5.568105	-1.093982	-0.086366
44	1	0	2.755935	0.693546	1.236626
45	1	0	1.671634	2.066566	1.036382
46	1	0	1.407655	0.890972	2.347217
47	1	0	-1.497286	0.836437	-2.305597
48	1	0	-2.560673	-0.581443	-2.374865
49	1	0	-0.838434	-0.807463	-2.300372
50	1	0	0.989174	-2.083070	2.607568
51	1	0	-2.872831	3.819461	0.704206
52	1	0	-7.461457	0.399146	-1.028337
53	1	0	-6.861909	-1.232007	-0.576536
54	1	0	-6.505936	-0.579000	-2.191869

Conformer 2

Center	Atomic	Atomic	Coordinates (Angstroms)		
Number	Number	Type	X	Y	Z
1	6	0	2.398439	-2.105256	1.540012
2	6	0	3.446914	-1.356207	0.772497
3	6	0	3.173176	-0.323220	-0.040514
4	6	0	1.750960	0.187786	-0.367744
5	6	0	0.654743	-0.764136	0.279480
6	6	0	1.125460	-1.286059	1.654759
7	6	0	1.593244	1.632676	0.187106
8	6	0	0.167928	2.177655	0.067795
9	6	0	-0.830034	1.290231	0.852509
10	6	0	-0.819985	-0.180352	0.349939
11	6	0	-2.217817	1.933435	0.720363
12	8	0	-2.962585	1.710085	-0.376659
13	6	0	-2.831258	0.515337	-1.197924
14	6	0	-1.468633	-0.200897	-1.060700
15	6	0	-4.058965	-0.326710	-0.960846
16	6	0	-5.153160	0.013059	-0.220844
17	8	0	-6.100017	-0.966379	-0.286079
18	6	0	-5.603523	-1.942956	-1.094052
19	6	0	-4.362464	-1.609987	-1.539552
20	6	0	-1.708476	-1.035287	1.283628
21	6	0	1.660672	0.190045	-1.922922
22	8	0	-2.604996	2.731081	1.550890

23	6	0	4.344298	0.397318	-0.651443
24	8	0	4.321681	1.517329	-1.128463
25	8	0	-0.485977	1.329182	2.231767
26	8	0	2.060963	-3.348675	0.897337
27	8	0	5.486926	-0.333035	-0.613143
28	6	0	6.652329	0.328689	-1.127322
29	1	0	2.783550	-2.316284	2.550916
30	1	0	4.480927	-1.662897	0.905258
31	1	0	0.589856	-1.651267	-0.365119
32	1	0	1.287131	-0.458849	2.352837
33	1	0	0.370781	-1.936313	2.098039
34	1	0	2.287584	2.295247	-0.333230
35	1	0	1.871406	1.643698	1.246433
36	1	0	-0.138080	2.274372	-0.980749
37	1	0	0.124164	3.185283	0.497553
38	1	0	-2.890570	0.907357	-2.220155
39	1	0	-1.601620	-1.243715	-1.370575
40	1	0	-0.800835	0.246754	-1.790737
41	1	0	-5.395733	0.874971	0.379727
42	1	0	-6.243929	-2.798342	-1.246624
43	1	0	-3.739109	-2.199903	-2.197591
44	1	0	-2.757397	-0.737387	1.200062
45	1	0	-1.651972	-2.094863	1.008762
46	1	0	-1.421155	-0.921683	2.328251
47	1	0	1.512757	-0.824760	-2.308675
48	1	0	2.571270	0.597585	-2.363644
49	1	0	0.847868	0.816710	-2.294588
50	1	0	-1.025507	2.053576	2.604751
51	1	0	2.887042	-3.819706	0.704237
52	1	0	7.464358	-0.391979	-1.024468
53	1	0	6.867148	1.234939	-0.554634
54	1	0	6.509026	0.599765	-2.176585

Conformer 3

Center	Atomic	Atomic	Coordinates (Angstroms)		
Number	Number	Type	X	Y	Z
1	6	0	-2.322470	2.468074	1.297931
2	6	0	-3.406754	1.698473	0.608150
3	6	0	-3.200053	0.557125	-0.068059
4	6	0	-1.810437	-0.072057	-0.323264
5	6	0	-0.662951	0.893555	0.212409
6	6	0	-1.100166	1.594835	1.516918
7	6	0	-1.718616	-1.446136	0.403344
8	6	0	-0.321490	-2.070230	0.363291
9	6	0	0.716976	-1.141520	1.037377

10	6	0	0.781873	0.249214	0.351094
11	6	0	2.074004	-1.859278	1.004490
12	8	0	2.849734	-1.787452	-0.089521
13	6	0	2.752468	-0.724590	-1.085182
14	6	0	1.419831	0.052831	-1.051030
15	6	0	4.011946	0.101709	-0.985127
16	6	0	4.246453	1.343469	-1.501560
17	8	0	5.537962	1.720024	-1.284790
18	6	0	6.139919	0.695380	-0.616546
19	6	0	5.262613	-0.322008	-0.411440
20	6	0	1.719273	1.165081	1.172509
21	6	0	-1.714137	-0.256991	-1.867965
22	8	0	2.408681	-2.574737	1.927742
23	6	0	-4.480694	-0.049428	-0.594702
24	8	0	-5.534951	0.550518	-0.680090
25	8	0	0.365562	-0.988228	2.406921
26	8	0	-1.909739	3.608976	0.522471
27	8	0	-4.380417	-1.357088	-0.937093
28	6	0	-5.599735	-1.965468	-1.395667
29	1	0	-2.699015	2.813945	2.274262
30	1	0	-4.424910	2.076859	0.670083
31	1	0	-0.560248	1.696320	-0.530220
32	1	0	-1.310145	0.863761	2.303568
33	1	0	-0.308567	2.246160	1.888158
34	1	0	-2.437778	-2.140832	-0.034516
35	1	0	-1.999957	-1.314868	1.453534
36	1	0	-0.020241	-2.314442	-0.662382
37	1	0	-0.327941	-3.016476	0.917050
38	1	0	2.791454	-1.280253	-2.029127
39	1	0	1.584597	1.038010	-1.500905
40	1	0	0.731157	-0.464969	-1.711725
41	1	0	3.639391	2.059971	-2.033307
42	1	0	7.176907	0.849256	-0.360042
43	1	0	5.449817	-1.257536	0.094483
44	1	0	2.751676	0.806841	1.128364
45	1	0	1.715736	2.185333	0.772248
46	1	0	1.431416	1.192800	2.222633
47	1	0	-1.462795	0.688181	-2.361214
48	1	0	-2.658980	-0.612469	-2.280981
49	1	0	-0.967856	-1.000534	-2.155608
50	1	0	0.868548	-1.684207	2.873757
51	1	0	-2.706924	4.097484	0.262468
52	1	0	-5.340489	-3.000686	-1.619420
53	1	0	-5.970092	-1.457572	-2.289709

54	1	0	-6.366512	-1.918422	-0.618407

Conformer 4

Center	Atomic	Atomic	Coordinates (Angstroms)		
Number	Number	Type	X	Y	Z
1	6	0	-2.322548	2.481477	1.280424
2	6	0	-3.408906	1.704655	0.602115
3	6	0	-3.203524	0.557522	-0.064598
4	6	0	-1.814090	-0.071743	-0.320436
5	6	0	-0.665818	0.901691	0.197658
6	6	0	-1.097168	1.612415	1.499129
7	6	0	-1.713883	-1.436354	0.421978
8	6	0	-0.316000	-2.057674	0.375850
9	6	0	0.728417	-1.121204	1.031423
10	6	0	0.782990	0.264596	0.332470
11	6	0	2.082254	-1.843956	0.984374
12	8	0	2.825466	-1.809145	-0.135990
13	6	0	2.742887	-0.731225	-1.111810
14	6	0	1.416116	0.060314	-1.070217
15	6	0	4.009039	0.078131	-0.994362
16	6	0	5.096214	-0.208918	-0.222425
17	8	0	6.082639	0.711668	-0.421094
18	6	0	5.618839	1.594659	-1.347479
19	6	0	4.359920	1.260326	-1.738314
20	6	0	1.719709	1.196240	1.136814
21	6	0	-1.726509	-0.274777	-1.863150
22	8	0	2.438583	-2.538703	1.915013
23	6	0	-4.485382	-0.056087	-0.579563
24	8	0	-5.539533	0.542947	-0.672130
25	8	0	0.393456	-0.957248	2.403320
26	8	0	-1.916653	3.617762	0.494705
27	8	0	-4.386267	-1.369097	-0.901296
28	6	0	-5.606899	-1.984605	-1.346608
29	1	0	-2.694636	2.833496	2.256297
30	1	0	-4.427234	2.082437	0.665133
31	1	0	-0.570323	1.698121	-0.552726
32	1	0	-1.301452	0.887000	2.292560
33	1	0	-0.305117	2.268077	1.861320
34	1	0	-2.435490	-2.137820	-0.000866
35	1	0	-1.985846	-1.293064	1.473106
36	1	0	-0.023067	-2.310845	-0.649980
37	1	0	-0.315881	-2.998687	0.938578
38	1	0	2.775397	-1.260703	-2.071346
39	1	0	1.594514	1.042967	-1.521186
40	1	0	0.721391	-0.451242	-1.729741

41	1	0	5.309327	-0.993960	0.485146
42	1	0	6.292878	2.394288	-1.614543
43	1	0	3.754711	1.785779	-2.464404
44	1	0	2.754923	0.848857	1.081143
45	1	0	1.698317	2.212980	0.728297
46	1	0	1.445114	1.229456	2.190404
47	1	0	-1.485809	0.666425	-2.369159
48	1	0	-2.671295	-0.642653	-2.265460
49	1	0	-0.976096	-1.015674	-2.146631
50	1	0	0.906446	-1.644856	2.871472
51	1	0	-2.716550	4.100782	0.232829
52	1	0	-5.348771	-3.023739	-1.552789
53	1	0	-5.978915	-1.492238	-2.248622
54	1	0	-6.372007	-1.923537	-0.568686
